# Radiolabelling of peptides with tetrazine ligation based on the inverse electron-demand Diels–Alder reaction: rapid, catalyst-free and mild conversion of 1,4-dihydropyridazines to pyridazines†

**DOI:** 10.1039/d3ra02807k

**Published:** 2023-07-26

**Authors:** Sofia Otaru, Tatu Martinmäki, Iida Kuurne, Andreas Paulus, Kerttuli Helariutta, Mirkka Sarparanta, Anu J. Airaksinen

**Affiliations:** a Department of Chemistry, Radiochemistry, University of Helsinki Finland; b Turku PET Centre, University of Turku Kiinamyllynkatu 4-8 FI-20520 Turku Finland anu.airaksinen@utu.fi; c Department of Chemistry, University of Turku Finland

## Abstract

Click chemistry reactions, such as the tetrazine ligation, based on the inverse-electron demand Diels–Alder (IEDDA), are chemoselective cycloaddition reactions widely used for chemical modifications and synthesis of biomolecule-based radiopharmaceuticals for positron emission tomography (PET). The reactions have potential also for pretargeted PET imaging. When used as a bioconjugation method in production of biomolecule-based radiopharmaceuticals, IEDDA-based tetrazine ligation has one significant drawback, namely the formation of a mixture comprising reduced metastable dihydropyridazines (DHPs) and oxidized cycloadducts. Conversion of the reduced DHPs to stable pyridazines requires oxidation, which is typically achieved by using oxidants or by photo-irradiated air-oxidation, both methods requiring added reagents or reaction times of several hours, not compatible with short-lived radionuclides. Here we report a mild, rapid, and catalyst-free conversion of the DHPs to pyridazines. In this study, a model peptide Tyr^3^-octreotide (TOC) was modified with polyethylene glycol (PEG) linkers and with *trans*-cyclooctenes (TCOs) for rapid IEDDA-mediated radiolabeling. Fluorine-18-labelled alkylammoniomethyltrifluoroborate ([^18^F]AmBF_3_) tetrazines were conjugated to the TCO-TOC analogs at room temperature for rapid synthesis of PET imaging agent candidates. The formed DHPs were successfully converted to the oxidized form, after heating the radiolabelled bioconjugates in aqueous solution (≥95% water) at 60 °C for a minimum of 10 minutes in the presence of air, resulting in one-pot back-to-back IEDDA reaction and DHP conversion. The water content of the reaction mixture was to be found critical for the coversion. Our finding offers a straightforward method for conversion of the metastable DHPs from the IEDDA-based tetrazine ligation to stable, oxidized pyridazines. The method is especially suitable for applications requiring rapid conversion.

## Abstract 2

Peptides were radiolabeled with [^18^F]AmBF_3_-Tz using IEDDA-based tetrazine ligation, producing radiopeptides in different oxidation states. In this study, the formed tautomers were rapidly (10 min) converted to the stable oxidized radiopeptides.

## Graphical abstract

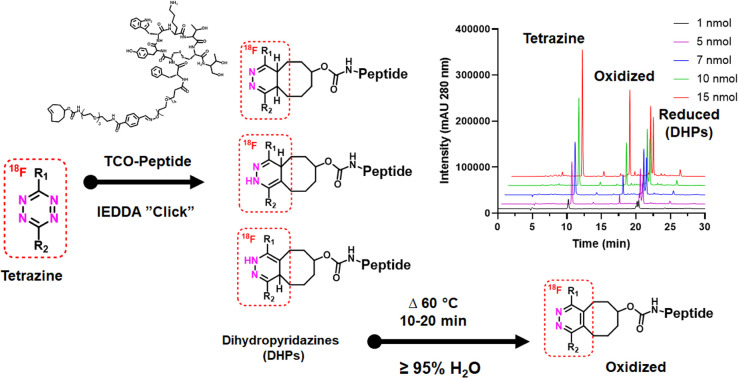

## Introduction

Positron emission tomography (PET) utilizes small compounds, peptides, nanoparticles or biomacromolecules radiolabelled typically with short-lived positron emitting radionuclides, that are administered into the study subject for the investigation of diseases and biological processes *in vivo*.^1–3^ However, in biomolecule-based radiopharmaceuticals, such as proteins and peptides, the sensitive target binding motifs might be compromised by the radiolabelling conditions, especially in the case of nucleophilic radiolabelling with fluorine-18. Milder methods, such as modular labelling using chemoselective, bioorthogonal click chemistry, would be preferred in-order to avoid the possible degradation or unintentional modification of the structure.

**Scheme 1:**
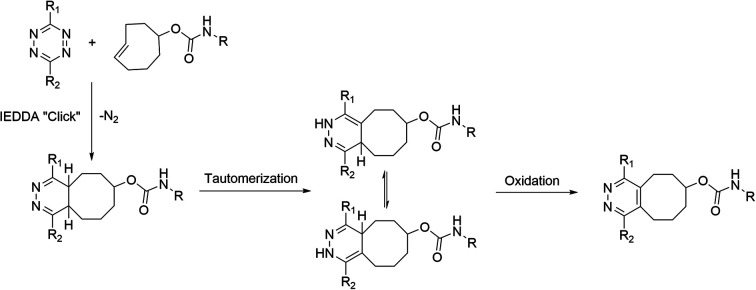
IEDDA cycloaddition followed by tautomerization of 4,5-DHP into 1,4-DHPs and subsequent oxidation of the bicyclic product to pyridazine. Substituents R_1_, R_2_; –H, –Ar, –Py, –Me.

The tetrazine ligation, based on the inverse electron-demand Diels–Alder (IEDDA) reaction, leverages the high reactivity of a *s*-tetrazine (Tz) with strained alkenes, such as *trans*-cyclooctene (TCO) forming a bicyclic compound as a product.^4^ Due to the unprecedented selectivity and reaction kinetics of the IEDDA-based tetrazine ligation, it has shown promise in *in vivo* imaging of targets, radiolabelling of sensitive constructs in physiological media including biomolecules, and has been applied in the study of physiological or pathological phenomena such as hypoxia in live cells *in* vitro.^5–11^ During the IEDDA cycloaddition 4,5-dihydropyridazines (4,5-DHPs) are formed, which then isomerize into 1,4-dihydropyridazines (1,4-DHPs). This results in the formation of several forms of the cycloaddition product.^12^ The metastable 1,4-DHPs are sluggishly oxidized (−2H) or aromatized into the stable pyridazine form (Scheme 1).^4,13^ The oxidation kinetics are slow, often requiring oxidizing reagents for obtaining the pyridazine as the primary product. Consequently, IEDDA-based tetrazine ligation is well suited for pretargeted imaging applications and for the radiolabelling of bulkier molecules, but analytical challenges are encountered when using tetrazine ligation for *in vitro* conjugation with small molecules due to the formation of several forms of isomers. Attempts for a practical and fast conversion of the DHPs to pyridazine have been reported.^13–15^ Recently Karaki *et al.* presented a catalyst-free oxidation under air by irradiating the reaction solution with ultraviolet light for up to 7 hours.^14^ The protocols reported to date have not yielded full conversion in minutes, rendering them better suited for radiolabelling applications using longer-lived radionuclides than short-lived ones, such as fluorine-18 (*t*_1/2_ = 109.8 min). If no steric hindrance is present in the structures the diene (1,2,4,5-tetrazine) and the dienophile (alkene or alkyne), can react both head-to-head or head-to-tail forming isomers.^16^ Hence, the cycloaddition produces also redox species in addition to the head-to-head or head-to-tail isomers.^17^ IEDDA reaction can be used as a bioconjugation method for radiolabeling of larger proteins, where the formation of the isomers and redox species is not evident during the radiosynthesis of large macromolecules, relying typically on size-exclusion chromatography (SEC) for analysis and QC of the final product, where these small differences arising from two protons cannot be detected. These chemical species are generally detected when utilizing the reaction for synthesis of radiopharmaceuticals with lower molecular mass but are considered irrelevant when IEDDA is used for pretargeting *in vivo*. However, awareness of this phenomenon is extremely important when evaluating performance and stability of new radiotracer candidates from *in vivo* samples. Selvaraj *et al.* reported they detected the oxidized form in *ex vivo* metabolic extract samples rapidly after administration of diphenyl-*s*-tetrazine-RGD and an ^18^F-labelled TCO derivative, comprising mainly of the reduced DHP forms.^18^ They detected both the oxidized and DHP analogues of their ^18^F-cRGD radiopeptide conjugate in blood, kidneys, liver, and urine 2 h post-injection. Both DHP and oxidized forms can undergo redox reactions and hence both can be considered metastable. Based on our investigation with AmBF_3_-TOC derivatives radiolabelled with fluorine-18 using the tetrazine ligation, we concluded that the oxidized pyridazine is extremely stable, resisting reduction to DHPs even *in vivo*.^19^

Recently, Litau *et al.* reported several side-products during radiometal labelling of peptides with IEDDA-based tetrazine ligation.^20^ In their study, DOTA-GA-Tz and NODA-GA-Tz were radiolabelled with gallium-68 and copper-64 prior to the IEDDA-mediated conjugation to the TCO-peptide, which resulted in formation of unknown side products. As a follow-up study from the same group, Damerow *et al.* reported the formation of DHPs after tetrazine ligation identified with HPLC-UV and MALDI-MS.^15^ The side-products formed again spontaneously and the full conversion of DHPs to pyridazine was successful for one of the four compounds studied in acidic conditions, resulting in evaluation of the other compounds as mixtures of both DHPs and pyridazine forms.

Radiopharmaceuticals need to meet strict quality requirements after production, the most significant being the purity of the final products. The cycloaddition between a 1,2,4,5-tetrazine and an alkene produces a mixture of redox species and structural isomers, each exhibiting different pharmacochemical properties potentially affecting biodistribution and affinity of the compounds to the target site. In this study, we report the characterization and rapid oxidation of the 1,4-DHP derivatives to the corresponding pyridazine after using IEDDA-based tetrazine ligation for radiolabelling of a model peptide. A clinically relevant somatostatin receptor 2 (SSTR2) targeting Tyr^3^-octreotide was selected as the model peptide. For a site specific TCO conjugation, the TOC peptide was functionalized with an aminooxy group and further functionalized either with TCO-aldehyde (TCO-CHO) or TCO-PEG_3_-aldehyde (TCO-PEG_3_-CHO) yielding two TOC peptide derivatives (TOC, 3*M*_w_; 1568 g mol^−1^ and 4*M*_w_; 1757 g mol^−1^). Two alkylammoniomethyltrifluoroborate (AmBF_3_) tetrazines (Tz), AmBF_3_-Tz (1)^14^ developed by us and a methyl-substituted AmBF_3_-PEG_4_-mTz (2) developed by Liu,^21^ were synthesized as prosthetic groups for IEDDA-based radiolabelling of the *trans*-cyclooctene (TCO) functionalized TOC peptides. The aim of this study was to elucidate the formation of redox species of the peptide (radio)conjugate during the IEDDA reaction, and to offer a straightforward protocol for converting the metastable reduced cycloaddition products to the oxidized form within the timeframe allowed by the physical half-life of fluorine-18 (*t*_1/2_ = 109.8 min).

## Results

### Synthesis of AmBF_3_ tetrazines

**Fig. 1:**
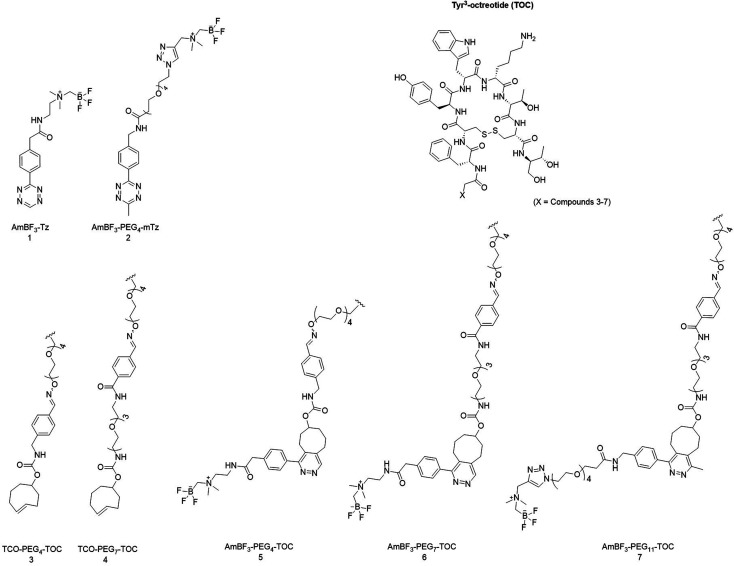
Chemical structures of Tyr^3^-octreotide (TOC), tetrazines 1 and 2, TCO-linkers attached to the TOC peptide (3, 4) and the IEDDA conjugates attached to the Tyr^3^-octreotide (TOC) derivatives (5, 6 and 7) after conjugation with 1 and 2 presented as the aromatized (oxidized) cycloaddition products. Structures 1 and 3–6 have been reported by us previously.^19^

Two AmBF_3_ tetrazines, 1 and 2, were synthesized with moderate to good yields (36–94%) (Fig. 1). The synthesis of AmBF_3_-Tz (1, ∼36% ≥ 99% purity) has been described earlier by our group,^19^ and AmBF_3_-PEG_4_-mTz (2) was synthesized with a previously published method by Liu *et al.*^22^ AmBF_3_-PEG_4_-mTz (2) was synthesised using a one-step protocol by copper(i) catalysed click chemistry (∼94% yield, ≥95% purity).

The chemical structures of the synthesized AmBF_3_ tetrazines are presented in Fig. 1. (See ESI for reaction conditions, synthesis schemes and characterization data; NMR, HPLC-DAD-ESI-MS, HRMS, HPLC data in Fig. S1–S22 and Tables S1–S5†).

### Synthesis of TCO-modified peptides and their IEDDA conjugates

The amino-oxy functionalized peptides were prepared by custom synthesis (CSBio, Kelly Ct, Menlo Park, CA, USA). The TCO-modified peptides were synthesized from the amino-oxy-TOC peptide. After the aminooxy-peptide and the TCO-aldehyde react by oxime formation, a double bond is formed, creating *E*- and *Z*-isomers of the peptide. The isomers were detected as a doublet peak of the TCO-TOC in 50 : 50 ratio in the HPLC chromatogram. TCO-modified peptides were purified with HPLC after the TCO-modification (Table 1 and Fig. 1). Instantly after the IEDDA conjugation of the tetrazines (1 or 2) with TOC several peaks were detected on the HPLC chromatogram (HPLC-RAD/DAD) with all tetrazine-to-peptide combinations (Table 1). The synthesized peptides were characterized with mass spectrometry (HPLC-DAD-ESI-MS or UHPLC-HRMS), and the purities verified with HPLC-DAD (209, 254 or 280 nm).

**Table 1:** Compound numbers assigned to tetrazines, TCO peptides and the peptide IEDDA conjugates in this study

Tetrazine	TCO-peptide	Peptide formed by IEDDA	Peptide name
1	3	5	AmBF_3_-PEG_4_-TOC
1	4	6	AmBF_3_-PEG_7_-TOC
2	4	7	AmBF_3_-PEG_11_-mTOC

### Radiolabelling of tetrazines and TCO-TOCs

Pyridazine HCl buffer (pH 2.0) was used for both radiolabelling and for elution of [^18^F]fluoride, similarly as reported earlier by Kwon *et al.*,^23^ from the PS-HCO_3_ ion exchange cartridge, instead of 0.9% NaCl, which is commonly used in isotopic exchange radiofluorinations of trifluoroborates. The tetrazines were radiolabelled by heating the precursors 1 or 2 in 0.9% NaCl together with pyridazine HCl buffer (pH 2) or the pyridazine HCl buffer alone (pH 2) at 85 °C. The radiochemical yields (RCY) and radiochemical purities (RCP) of the tetrazine prosthetic groups are presented in Table 2. See ESI Table S6† for the pyridazine HCl buffer recipe.

**Table 2:** Radiochemical yields (RCYs), radiochemical purities (RCP) and molar activities of radiotracers. Values are decay corrected to the start of synthesis

Compound	RCY (%) from [^18^F]fluoride	RCP (%)	RCY (%) from [^18^F]AmBF_3_-tetrazine	Molar activity (*A*_m_) (GBq μmol^−1^)
[^18^F]1	19 ± 9% (*n* = 4)	≥99%	Not applicable	15.4 ± 9.2 (*n* = 14)
[^18^F]2	20.1% (*n* = 1)	≥95%	Not applicable	n. d.
	22.3 ± 11.4% (*n* = 3)^a^			
[^18^F]5	3.3 ± 1.7% (*n* = 3)	≥95%	19.3 ± 11.6% (*n* = 3)	2.8 ± 1.8 (*n* = 3)
[^18^F]6	5.1 ± 3.4% (*n* = 5)	≥99%	21.4 ± 13.5% (*n* = 4)	6.0 ± 3.4 (*n* = 13)
[^18^F]7	0.9 ± 1.0% (*n* = 2)	≥99%	n. d.	0.7 ± 0.7 (*n* = 3)

a Incorporation yield, n. d. = not determined.

### IEDDA conjugation between TCO-TOC and [^19^F/^18^F]AmBF_3_-tetrazines: detection of unknown species

TCO-peptides (3 or 4) were conjugated with [^19^F/^18^F]AmBF_3_-tetrazines (1 and 2) resulting in a complex mixture of products detected with both DAD and radio-HPLC analysis. Upon conjugation of TCO-PEG_7_-TOC (4) with [^19^/^18^F]AmBF_3_-Tz ([^19^/^18^F]1) forming [^19^F/^18^F]6, the peak cluster (*t*_R_ = 20.2–20.6 min) was accompanied by a small peak eluting earlier in the chromatogram (ESI Fig S14,† radio-HPLC). After IEDDA conjugation, the cluster of peaks (peptide 6, *t*_R_ = 20.2–20.6 min) was collected and left to room temperature for 18 hours. A sample was injected to HPLC-DAD and UHPLC-HRMS, and the cluster of peaks (*t*_R_ ≈ 20 min, HPLC) had converted to one single peak (peptide 6, *t*_R_ = 17 min), and it was apparent that once left to incubate at room temperature, the total conversion (from *t*_R_ ≈ 20 min to *t*_R_ = 17 min) was achieved overnight. The (radio)peak arising from compound [^18^F]6(a, b) (*t*_R_ = 20 min) was collected and re-injected to HPLC, and in the second HPLC run, a more polar radiopeak was again detected (peptide 6, *t*_R_ = 17 min), accompanied by [^18^F]6(a, b) (peptide 6, *t*_R_ = 20 min). The polar radiopeak (peptide 6, *t*_R_ = 17 min) grew as a function of time, irrespective of the HPLC purification of the radiopeak and it was apparent this so far unknown compound converted from [^18^F]6(a, b) (*t*_R_ = 20 min) to the polar radiopeak (peptide 6, *t*_R_ = 17 min) likely to the oxidized tautomer of [^18^F]6(a, b)-DHP.

### UHPLC-HRMS characterization of the IEDDA-based tetrazine ligation products and characterization of the oxidized form

After the overnight tetrazine ligation of 4 and 1 at room temperature, yielding peptide-conjugate 7, the crude sample was subjected to ultra-high-performance liquid-chromatography high-resolution mass spectrometry (UHPLC-HRMS). This confirmed, that the polar (*t*_R_ = 17 min) compound [^18^F]6(a, b) corresponded with the oxidized form of the peptide after the IEDDA cycloaddition (*t*_R_ = 4.93 min in the UHPLC system), detected in the total ion chromatogram (TIC) (ESI Fig. S15†).

The crude reaction mixture of AmBF_3_-PEG_4_-mTz (2) and peptide 4, resulting in the peptide conjugate AmBF_3_-mTz-PEG_11_-TOC (7), was analysed with UHPLC-HRMS immediately after the IEDDA reaction. All the expected analogues of 7 were detected as protonated DHP species, based on the extracted ion chromatograms (EICs) (ESI Fig. S17–S22†). The methyl tetrazine eluted at 4.81 min (ESI Fig. S16†). The oxidized AmBF_3_-PEG_11_-m-TOC(ox.) eluted at 4.90 min (ESI Fig. S17–S18†). The reduced AmBF_3_-PEG_11_-m-TOC(DHP) was detected at *t*_R_ = 5.05–5.07 min as a doublet peak, indicative of two main DHP analogues (ESI Fig. S19–S22†). The elemental compositions together with calculated and found masses with measured mass error and retention times are tabulated in Table 3.

**Table 3:** UHPLC-HRMS analysis of compounds

Compound	Ion	Elemental composition	Calc. (*m*/*z*)	Found (*m*/*z*)	Mass error (*Δ*, ppm)	*t*_R_ (min)
2	[M − F]^+^	C_27_H_41_O_5_N_9_BF_2_	620.32863	620.32990	2.04132	4.81
7(ox.)	[M + 2H]^2+^	C_112_H_162_O_28_N_20_BF_3_S_2_^2^	1183.56715	1183.56799	0.71097	4.90
7(DHP)	[M + 2H]^2+^	C_112_H_164_O_28_N_20_BF_3_S_2_^2^	1184.57498	1184.57642	1.21504	5.05
7(DHP)	[M + 2H]^2+^	C_112_H_164_O_28_N_20_BF_3_S_2_^2^	1184.57498	1184.57568	0.59674	5.07

The IEDDA conjugated peptide 6 was detected, after overnight incubation at room temperature as mainly a singlet peak (*t*_R_ = 4.93 min) in the UHPLC-HRMS studies. It was determined that the singlet more polar [^19^F/^18^F]6(a, b) peak (*t*_R_ = 17 min HPLC, *t*_R_ = 4.93 min UHPLC) was mainly in the oxidized (−2H, ox.) form, based on the total ion chromatogram (TIC) (ESI Fig. S15†).

### Optimization of the conditions for rapid conversion of reduced peptides to oxidized peptides

**Fig. 2:**
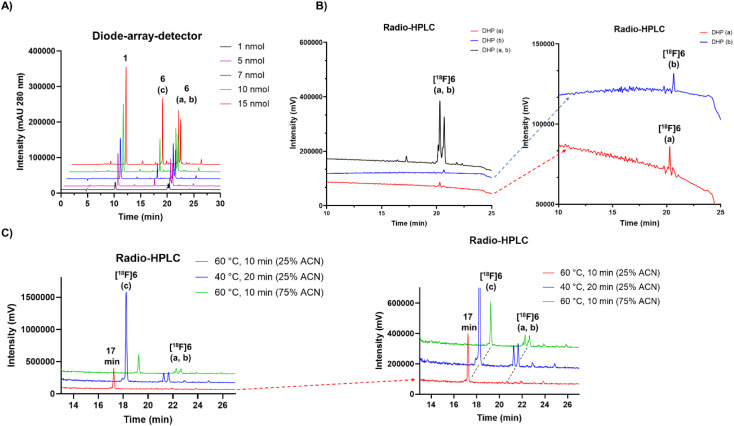
(A) HPLC-DAD (280 nm, for HPLC method, see ESI Table S2†) chromatogram of IEDDA conjugation reaction between compound 1 and peptide with increasing molar amounts of 4, yielding peptide conjugate 6. The unreacted compound 1 used in excess is detected at *t*_R_ = 10 min. The analogues a and b of the peptide 6 are detected at *t*_R_ = 20.2–20.6 min (a, b) accompanied by an additional peak at *t*_R_ = 17 min (c), hypothetically of 6 as well. (B) Radio-HPLC chromatogram of HPLC purified [^18^F]6 at *t*_R_ = 20.2–20.6 min, together with the chromatograms of HPLC separated proposed isomers a (red trace) and b (blue trace) of the radiopeptide. (C) Radio-HPLC chromatogram of [^18^F]6 incubated in different reaction conditions revealing two analogues [^18^F]6a and b at *t*_R_ = 20.2–20.6 min, and a third [^18^F]6c at *t*_R_ = 17 min. The optimal reaction conditions resulting in ≥97% of [^18^F]6c after incubation at 60 °C for 10 min with minimum of 75% of water and maximum of 25% ACN (20–60 μL total volume and 2–7.5 nmol of TCO-PEG_7_-TOC).

Protic solvents in acidic reaction conditions at various temperatures were used in-order to investigate the conditions that might influence the formation of the oxidized form from DHPs or that might slow down the process. In buffered solutions at pH range of 3.8–6.8 the formation of the oxidized analogue was nearly fully prevented, whereas once diluted with ultrapure water (≥95% v/v), the total conversion of the DHPs into a single compound was achieved. The full conversion required only simple heating in diluted solution at 60 °C for minimum of 10 minutes in the presence of air (ESI Table S7†). The (radio)peaks of [^19^F/^18^F]6(a, b) (peptide 6, *t*_R_ = 20 min) were successfully converted (≥97%) to the more polar compound (peptide 6, *t*_R_ = 17 min) by incubation in increasing amounts of water in acetonitrile (Fig. 2C). When incubating in 75% (v/v) ACN in water for 10 min at 60 °C, the conversion to polar (peptide 6, *t*_R_ = 17 min) compound was 59% (ESI Table S7†). In 25% (v/v) ACN in water at 40 °C a conversion of 86% was achieved in 20 minutes. The highest conversion of ≥97% to *t*_R_ = 17 min was achieved in 25% (v/v) ACN in water at 60 °C (Fig. 2C), which was later optimized to 95 : 5% water : ACN and 20 min incubation time at 60 °C, for a repeatable, complete and reliable conversion of [^18^F]6(a, b) fully to the polar radiopeak (peptide 6, *t*_R_ = 17 min).

**Fig. 3:**
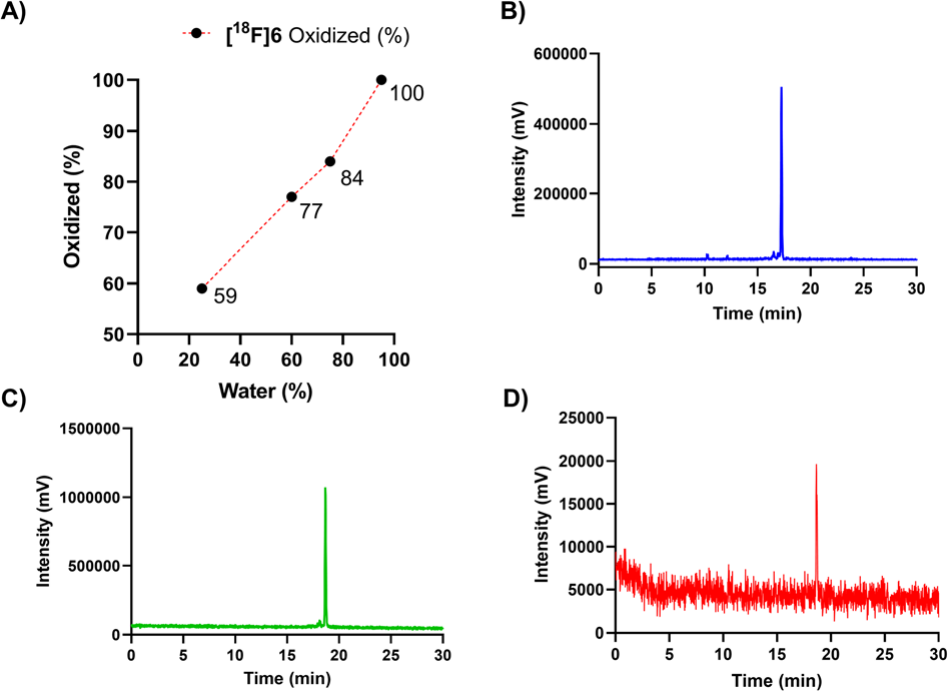
(A) Dependence of oxidation (% of the oxidized form in the reaction mixture) on the ratio of water-to-acetonitrile (water%) for peptide [^18^F]6, during incubation with a solution comprising of 25, 60, 75 and 95% (v/v) of water at 60 °C (*n* = 1). Radio-HPLC chromatograms (for HPLC method, see ESI Table S2†) of the oxidized forms of the peptide cycloadducts (B) [^18^F]6*t*_R_ = 17.2 min, (C) [^18^F]5*t*_R_ = 18.5 min and (D) [^18^F]7*t*_R_ = 18.6 min after incubation in ≥95% (v/v) water at 60 °C yielding full conversion.

Adjustment of the reaction medium pH or using a buffer solution at pH in the range of 3.75–6.8 prevented the full conversion of the [^18^F]6(a, b) peaks to one polar (*t*_R_ = 17 min) compound. The selection of solvent, together with heating for 10 min to 60 °C promoted the oxidation conversion dramatically, which was facilitated the most when water was the major solvent, yielding full conversion in ≥95% (v/v) water (Fig. 3A).

A heating step of 20 min was later applied for upscaling the radiosynthesis, as a precautionary measure to ensure total conversion irrespective of the reaction volume, even at reaction volumes of ≥500 μL. The radio-HPLC chromatograms of the fully converted purified radiopeptides [^18^F]5, [^18^F]6 and [^18^F]7, are presented in Fig. 3B–D and radiochemical yields in Table 2.

### The redox state affects lipophilicity of the IEDDA cycloaddition products

**Fig. 4:**
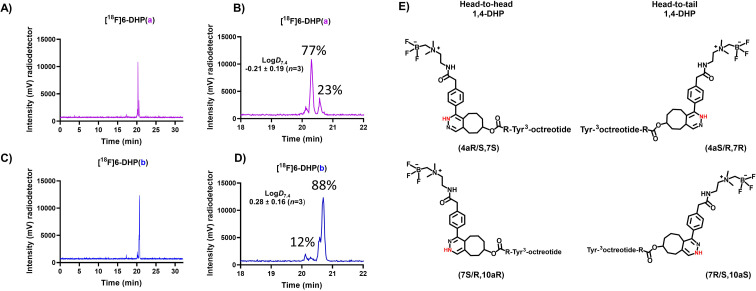
(A–D) HPLC (radio-HPLC) chromatograms (for HPLC method, see ESI Table S2†) of analogues of [^18^F]6(DHP(a, b), DHP(a) and DHP(b)) after HPLC separation of the fractions for log *D*_7.4_ evaluation, together with the (E) chemical structures of head-to-head and head-to-tail conformations of 1,4-DHPs.

A sample of the radiolabelled DHP-derivative [^18^F]6-DHP(a, b) was injected to HPLC and the analogues [^18^F]6-DHP(a) and [^18^F]6-DHP(b) separated with a fraction collector for log *D*_7.4_ determination (Table S4†). Analog [^18^F]6(ox.) was obtained as 100% pure due to good HPLC separation, but analogues [^18^F]6-DHP(a) (77% purity) and [^18^F]6-DHP(b) (88% purity) were obtained as a mixture of the two DHP analogues and evaluated as such (Fig. 4). The log *D*_7.4_ values of the separated analogues revealed that the oxidized form of [^18^F]6(ox.) was most hydrophilic (log *D*_7.4_ = −0.73 ± 0.12, *n* = 4), and the analogue eluting last from the reversed phase column, [^18^F]6-DHP(b), exhibited the highest lipophilicity (log *D*_7.4_ = 0.28 ± 0.16, *n* = 3), as the chromatographic separation also indicated. The lipophilicity of [^18^F]6-DHP(b) influenced the log *D*_7.4_ value of the cluster [^18^F]6-DHP(a, b) by increasing it near zero (log *D*_7.4_ = −0.04 ± 0.02, *n* = 3).

### Cell uptake of oxidized Tyr^3^-octreotides [^18^F]6 and [^18^F]7 in AR42J cells

**Fig. 5:**
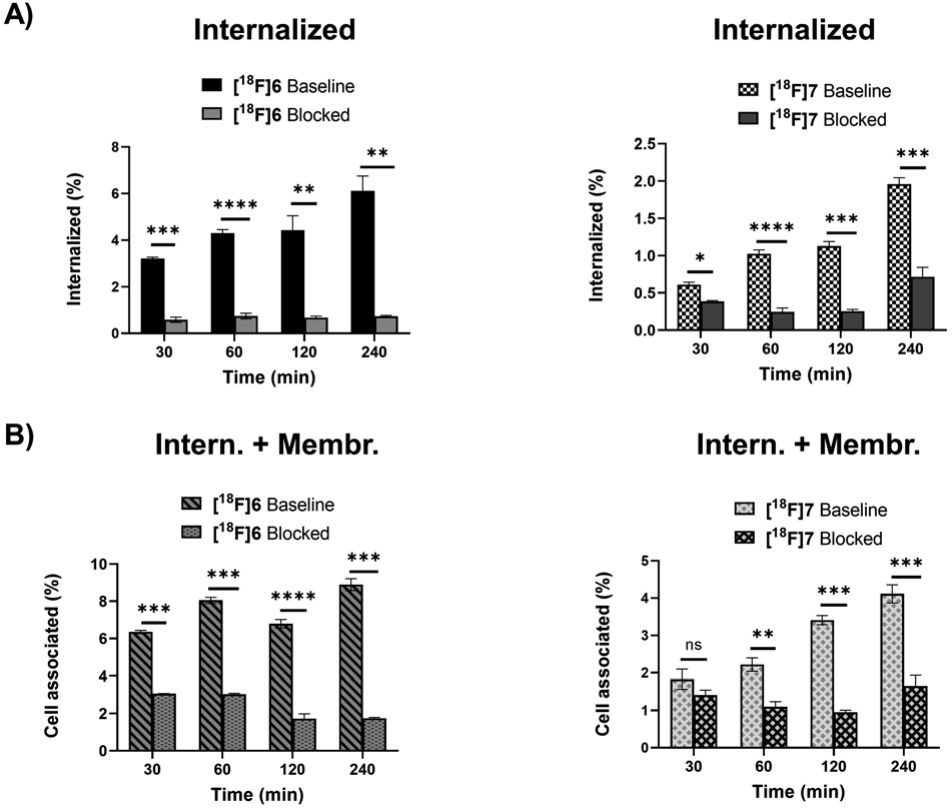
AR42J cell uptake of oxidized Tyr^3^-octreotide peptide tracers [^18^F]6 and [^18^F]7 demonstrating specific uptake in both internalized and total cell associated fractions (=internalized + membrane bound) that was blocked by co-incubation of non-modified octreotide. Intern.; internalized, Membr; membrane bound. The data is presented as mean ± standard deviation of cell uptake (%) from the total added radioactivity. Statistical significance *p < 0.05, **p < 0.005, ***p < 0.001, ****p < 0.0001. (Panel A) Internalized fraction. (Panel B) Cell associated = membrane bound and internalized fractions.

The radiolabelled peptides were studied in SSTR2-expressing rat pancreatic adenocarcinoma AR42J cells to investigate their internalization and specificity of the binding. After conversion of all tautomers to the pyridazine form, both peptides [^18^F]6 and [^18^F]7 bound specifically to the tumour receptors and were internalized by the cells (Fig. 5). The oxidized pyridazine peptides of [^18^F]6 and [^18^F]7 demonstrated specific uptake in AR42J cells, which was successfully blocked by adding the non-radiolabelled competitor octreotide in excess (Fig. 5).

## Discussion

The aim of this study was to investigate cycloaddition products after using IEDDA-based tetrazine ligation for radiolabelling of TCO-modified peptides and to develop a protocol for rapid oxidation of the DHPs to pyridazines. The reduced DHP cycloaddition products were slowly but spontaneously oxidized, and the oxidation was further accelerated by heating the reaction mixture in aqueous solvent with >75% water at 60 °C in the presence of air. In a previous study by Karaki *et al.*, the reaction kinetics were accelerated successfully by UV light for several studied DHP derivatives, affording relatively rapid transformation of a DHP cycloadduct between 3,6-di(2-pyridyl)-1,2,4,5-tetrazine and TCO yielding the pyridazine in 76% conversion in CH_2_Cl_2_ under LED light (*λ* = 365 nm) in approximately 5 hours.^14^ They showed that near full conversion of DHP to pyridazine (96%) was achieved in 7 hours, under air atmosphere and while kept dark using CH_2_Cl_2_ as a solvent. The authors hypothesized that the reaction mechanism proceeded by a singlet oxygen reacting with 1,4-dihydropyridazine, yielding hydrogen peroxide and pyridazine. Furthermore, the tautomeric conversion, where the DHP intermediate plays a key role, has been applied for an extremely fast Tz-TCO click-to-release scheme with the attempt to deliver payloads, with Tz-TCO ligation taking place at a pH ≤ 6 and by driving the compounds to react in a “head-to-head” instead of “head-to-tail” configuration by introduction of bulky N–Me group to the TCO that reacts with the methyl-tetrazine, resulting in the desired product.^16^

In this work, the TCO-peptides were conjugated with AmBF_3_ tetrazines, resulting in tautomers of [^18^F]6 amenable to chromatographic separation. After lipophilicity determination, it was concluded that all derivatives of [^18^F]6 or their mixtures (DHP analogues) demonstrated slightly different lipophilicities. It was shown, that the peptide cycloadduct itself was the origin of the DHP compounds, a phenomenon described in earlier studies for the IEDDA-based tetrazine ligation. It was apparent that the cycloaddition created several derivatives at two redox states (Scheme 1, Fig. 2 and 4), in addition to the “head-to-head” and “head-to-tail”. In a similar study describing several formed IEDDA cycloaddition products by Litau *et al.*, the radiolabelled side products were not detected on the UV trace of HPLC, but only on the radiodetector, contrary to our findings.^20^ Whether the detected radiopeaks arose from the DHPs and pyridazine forms of the bicyclic products or whether these redox species were at all detected during the study was, however, left undisclosed.

The chromatographic behaviour of the cycloadduct analogues in our study correspond with the ones report by Selvaraj *et al.*, where the DHPs were more retained, and the elution order was the same on a reverse-phase column.^18^ In our work reported herein, the peak eluting fastest on the C18 column was determined to be the oxidized peak, followed by the more retained DHPs. This is in accordance with the differences in lipophilicity determined with the shake-flask method.

However, the identification of 1,4- and 4,5-dihydropyridazines from the peak clusters would require MS/HRMS studies or 2D NMR experiments, which fell beyond the scope of this study. Due to the spontaneous conversion of the reduced DHP analogues to the oxidized pyridazine analogue, it was obviously rational to study the most stable oxidized form in a cell uptake assay. The oxidized forms of the two selected radiolabelled TOCs [^18^F]6 and [^18^F]7 demonstrated specific cell uptake in AR42J cells, which was efficiently blocked by co-incubation with an excess of non-labelled octreotide, indicating the receptor-mediated uptake was selective and not disrupted by any of the manipulations applied during the conversions.

The IEDDA-based tetrazine ligation is extremely efficient at room temperature and requires no heating to reach completion. The formation of several tautomers during the cycloaddition result in a complex mixture and the conversion of the DHPs to pyridazine reaches completion at room temperature but is slow, as demonstrated by us (18 h) and by Navarro *et al.* (7 days).^13^ The conversion can be sped up and the objective of this study was to find simple conversion conditions. Two mild methods, either heating at 60 °C for 10–20 minutes in aqueous solution with the presence of air, or incubation overnight at room temperature, were recognized suitable for converting the reduced species to the oxidized. The latter incubation method is the preferred method when applying the IEDDA for heat-sensitive constructs with long-lived radionuclides or for non-radioactive constructs. For rapid radiolabelling protocols with short-lived radionuclides, an overnight incubation would not be practical and heating to 60 °C for 10–20 min was determined sufficient for the conversion irrespective of the tetrazine used in this study. The hypothetical participation of atmospheric air can be deducted from previous literature, but no direct correlation was demonstrated in our tests. Since the application of the TCO peptide in water was done by an external syringe line, followed by air, and the air in the line was the only source of atmospheric air in our setup. The presence of pyridazine arising from the pyridazine HCl buffer and the excess of the tetrazine in the dilute reaction mixture were hypothesized to have, at best, a catalytic influence as sacrificial electron donors/acceptors on the oxidation mechanism, if any. A direct correlation with the water content in the solution was proven, but the specific mechanism was left unclear in this study and warrants further investigation.

## Experimental

### Reagents and equipment

Tetrazines were synthesized from commercial reagents. AmBF_3_-Tz (1) was synthesized as reported previously by us.^19^ Methyl-tetrazine-PEG_4_-azide was purchased from Conju-Probe LLC (San Diego, CA, USA). TCO-CHO was synthesized from commercial starting materials as reported earlier by us.^19^ 4-(aminomethyl)benzaldehyde was purchased from Enamine (Monmouth, NJ, USA). Tetrazine NHS ester (BroadPharm, San Diego, CA, USA), iodomethylboronic acid pinacol ester (Enamine, Riga, Latvia), TCO-*N*-hydroxysuccinimide (NHS) ester (Jena Bioscience, Jena, Germany) and TCO-PEG_3_-aldehyde (Conju-Probe, San Diego, CA, USA) were used as received. Custom synthesized aminooxy-functionalized peptides were purchased from CSBio (Kelly Ct. Menlo Park, CA, USA). Rat pancreatic tumour cell line AR42J (ATCC CRL-1492), expressing SSTR2, was obtained from American Type Culture Collection (Manassas, VA, USA). Dry acetonitrile (DNA synthesis quality, max. 10 ppm H_2_O) was purchased from Sigma Aldrich (Supelco). Pyridine, THF, ACN and DMSO, all anhydrous, were purchased from Sigma-Aldrich (St. Louis, Missouri, USA). Reactions done in argon atmosphere were prepared by heating all the glassware in an oven overnight and cooled with argon gas prior to use. Sep-Pak C18-Light cartridges were purchased from Waters and PS-HCO_3_-cartridges (Macherey-Nagel™ Chromafix™) from Fisher Scientific (Waltham, MA, USA). No-carrier-added ^18^F-fluoride was produced in-house with an IBA Cyclone 10/5 medical cyclotron by a ^18^O(p,n)^18^F reaction from ^18^O-enriched water (≥97%) purchased from Rotem Industries Limited (Arava, Israel) or Campro Scientific (Berlin, Germany). The compounds were analysed by nuclear magnetic resonance spectroscopy (NMR, 400 MHz Bruker Avance NEO NMR spectrometer), radio-thin-layer-chromatography (radio-TLC, silica; TLC silica gel 60 F_254_, reverse-phase; Supelco TLC silica gel 60 RP-18 F_254S_) and radio-high-performance liquid chromatography (radio-HPLC) utilizing diode-array-detector (DAD) and radiodetection. The radioactivity was measured with a Wizard 3′′ gamma counter (PerkinElmer, Turku, Finland). The radiolabelling experiments were executed in a hot cell with a semiautomated synthesis unit by DM Automation (Nykvarn, Sweden).

### Synthesis of tetrazines 1 and 2

AmBF_3_-Tz (1) was synthesized as published previously by our group.^19^ AmBF_3_-alkyne precursor was synthesized as described earlier by Liu *et al.*^22^ (ESI Scheme S1†). AmBF_3_-alkyne was conjugated with the methyl-tetrazine-PEG_4_-azide forming AmBF_3_-PEG_4_-mTz (2) in one step (ESI Scheme S2†) as reported earlier by Liu.^21^

### Synthesis of *trans*-cyclooctene aldehyde (TCO-CHO)

TCO-CHO was synthesized according to our previous publication.^19^ The chemical structures of TCO-aldehydes TCO-CHO and the commercially available TCO-PEG_3_-CHO are presented in the ESI Fig. S10.†

### *Trans*-cyclooctene (TCO) modification of peptide

The aminooxy peptide was functionalized with TCOs by a protocol described in ESI.† Briefly, the peptide (1 eq.) was dissolved into 600 μL of 0.3 M anilinium acetate buffer (pH 4.6). TCO-aldehyde (1.5 eq.) without a PEG-linker or with PEG_3_-linker was dissolved in chloroform (17 μL) and added into the stirred peptide solution dropwise. The reaction monitoring was done with HPLC coupled to a DAD detector at wavelength of 280 nm, and the modified peptide (5, 6 or 7) was purified with semipreparative HPLC and were analysed with HPLC-DAD-ESI-MS or UHPLC-HRMS, and the purities verified by HPLC-DAD (209 or 254 nm).

### IEDDA cycloaddition between TCO-peptides and tetrazines

TCO-modified peptides (50 nmol) were mixed with the tetrazines (1 or 2, 100 nmol). The reaction mixture was diluted to constitute approximately 95% of water (500 μL volume) and the reaction mixture was heated at 60 °C for 10–20 minutes. The product was purified with Sep-Pak SPE C18 cartridge and analysed with HPLC.

### Radiolabelling of AmBF_3_ tetrazines

The tetrazine 1 was added with 5 μL of dry ACN to the bottom of a 2.5 mL conical polypropylene tube containing 10 μL of pyridazine HCl buffer (pH 2.0). Methyl-tetrazine 2 was dissolved in a conical polyproplylene tube to 20 μL of pyridazine HCl buffer (pH 2) without ACN. [^18^F]Fluoride was unloaded from the cyclotron and trapped with a PS-HCO_3_ (cartridge Macherey-Nagel™ Chromafix™) from target water, and eluted with 100 μL pyridazine HCl buffer to the reaction vial preheated to 85 °C containing the tetrazine. The reaction mixture was heated for 15 minutes while an argon gas flow was applied, until 600 μL of ultrapure water was added with an external syringe line to quench the reaction, followed by 1 mL of air. Either protocol A (SPE-purified [^18^F]F-labelled tetrazines for IEDDA) or protocol B (crude [^18^F]F-labelled tetrazines for IEDDA) was applied from here on.

#### Protocol A

Radiolabelling of tetrazines [^18^F]1 and [^18^F]2 (range 100–200 nmol). The crude reaction mixture was further diluted with 4 mL of ultrapure water and loaded on a SPE C_18_ cartridge in the hot cell by semiautomated syringe pump setup attached to the synthesis unit. The cartridge was washed with 40 mL water, dried with 10 mL of air, and eluted with dry ACN (∼300 μL) through an external line into a lead-shielded vial. The products were analysed by HPLC with co-injection of the reference compounds.

#### Protocol B

Radiolabelling of peptides by IEDDA. The temperature was adjusted to 60 °C, gas flow was turned off and the TCO-modified peptide (50 nmol, in 500 μL of ultrapure water) was added through an external line to the reaction vial containing the crude radiolabelled tetrazine and approximately 1 mL air was added through the external line. After 10–20 minutes at 60 °C (95 : 5 v/v H_2_O : ACN) the reaction mixture was diluted with 8 mL of ultrapure water applied to two stacked SPE C18 cartridges by a remote controlled syringe. The C18 cartridges were washed with ultrapure water (40 mL), 20% EtOH (3 mL) and the [^18^F]AmBF_3_-labelled peptide was eluted with 400 μL of ethanol and 400 μL of 10× PBS (pH = 7.4) and diluted with ultrapure water to 1× PBS. The products were analysed with HPLC.

### Chromatographic analysis of peptide conjugates

The peptide derivatives were analysed by high-performance liquid-chromatography with diode array-detection (HPLC-DAD), radiodetection (HPLC-RAD), and by ultra-high-performance liquid chromatography high-resolution mass spectrometry (UHPLC-HRMS) or HPLC coupled to DAD and electrospray-ionization mass spectrometry (HPLC-DAD-ESI-MS) detectors for accurate mass or elemental composition determination.

### UHPLC-HRMS

Selected compounds were analysed by UHPLC Thermo Scientific Dionex Ultimate 3000 ultrahigh performance liquid chromatography system (Germering, Germany) which was coupled to a Thermo Scientific Orbitrap Fusion mass spectrometer (San Jose, CA, USA). Ionization was done with a heated electrospray ionization (HESI) source operated in positive ionization mode (HESI^+^). The scan range was set at 120–1200 *m*/*z* and 100–2000 *m*/*z*. The acquired data was processed with Xcalibur™ workstation (Thermo Fisher Scientific, Waltham, MA, USA). The peptides were analysed for the detection of their exact masses and proposed elemental compositions, including mass errors. The mass error was set at 3 ppm. Used elution gradient is presented in Table S1.†

### HPLC-DAD-ESI-MS analysis

Selected compounds were analysed with Agilent Technologies 1260 Infinity HPLC-DAD system with Agilent Technologies 6120 Quadrupole LC/MS detector. Ionization was executed with HESI^+^, at a scan range of scan range 100–2000 *m*/*z*. Data was processed with OpenLAB CDS Workstation. The gradient that was used for HPLC studies is presented in Table S2.†

### HPLC-RAD/DAD and HPLC-RAD/UV

The HPLC instruments were equipped with either two channel UV/VIS or a PDA detector, and a NaI(Tl) scintillation detector for detection of radioactivity. The gradient that was used for HPLC studies is presented in Table S2.†

### Lipophilicity and cell uptake assay in AR42J cells

The HPLC-separated analogues of [^18^F]6 (a, b; DHP analogues c; oxidized analogue) were subjected to log *D*_7.4_ studies by the shake-flask method detailed in the ESI.† The protocols used for log *D*_7.4_ evaluation and cell uptake assays have been previously published by our group.^19^ Briefly, the oxidized (*t*_R_ = 17 min) and the co-eluting DHP (*t*_R_ = 20 min) forms of [^18^F]6 were separated from each other by radio-HPLC affording three fractions; oxidized (fraction 1), DHP a (fraction 2) and DHP b (fraction 3), which were diluted into the cell media for uptake studies following the protocol given in the ESI.† Briefly, the radiotracer was purified by Sep-pak C18 cartridge and its cell uptake investigated in AR42J cells. The C18 purified tracer (700 μL) was added to CO_2_ independent medium (26.0–30.0 mL). The medium was divided in half for blocking and non-blocking formulations, both in 13.0–15.0 mL volume with 2.6% v/v EtOH. 65 μL of 1 mg mL^−1^ octreotide solution in 1× PBS was added to the blocking medium (concentration 4.9 μM). 1.0 mL of the prepared non-blocking or blocking solutions were applied on 1 million AR42J cells on a 6-well plate, in triplicates (*n* = 3) in each timepoint (30, 60, 120 and 240 min) per condition. [^18^F]6 was used in 0.04 nmols per well, and for blocking a 220 × excess (8.8 nmol) of octreotide was added to the formulation. The peptide [^18^F]7 was used in 0.9 nmols and was applied per well to non-blocking and for blocking conditions, also 377 nmol of octreotide was added.

### Statistical analysis

The statistical significance between averages from different groups were evaluated using Welch's *t*-test on GraphPad Prism (version 9.1.1) software (GraphPad Software, LLC). Statistical significance was set at *p < 0.05, **p < 0.005, ***p < 0.001, ****p < 0.0001.

## Conclusions

In this study, we report a rapid, mild, and facile method for converting dihydropyridazine cycloaddition products to pyridazine during IEDDA, in only 10 minutes. The integrity of the most stable (oxidized) cycloaddition analogue was further confirmed to display receptor-mediated uptake in AR42J cells by evaluating two TOCs [^18^F]6 and [^18^F]7, and the uptake was efficiently blocked by competitor octreotide. Furthermore, the *ex vivo* biodistribution and PET/CT imaging studies conducted in an earlier cohort by us, demonstrated that the TOC peptide [^18^F]6 was able to visualize the tumour *in vivo*, and the tumour uptake was as efficiently blocked. Based on our findings, one of the caveats identified for IEDDA-based tetrazine ligation can been overcome easily and a plethora of IEDDA mediated *in vitro* conjugations, radiolabelled or not, will be reinvestigated and potentially improved with the “dihydropyridazine to pyridazine”-conversion protocol reported herein. As demonstrated, IEDDA-based tetrazine ligation has great potential as a sophisticated, rapid and chemoselective means even for *in vitro* construction of bioconjugates in various applications. Here, we demonstrated with a clinically relevant peptide derivative, a simple method requiring no oxidants or additional complicated apparatus providing full conversion of reduced DHPs to one oxidized form. The findings reported in this study are of importance for both *in vitro* structural modifications by IEDDA and for improving the success of *in vitro* “click” conjugation strategies based on IEDDA-mediated tetrazine ligation.

## Author contributions

Sofia Otaru; writing – original draft, writing – review & editing, formal analysis, investigation, conceptualization, visualization. Tatu Martinmäki; writing – review & editing, formal analysis, investigation. Iida Kuurne; writing – review & editing, formal analysis, investigation. Andreas Paulus; writing – review & editing, formal analysis, investigation, conceptualization. Kerttuli Helartta; writing – review & editing, supervision, resources. Mirkka Sarparanta; writing – review & editing, supervision, resources. Anu J. Airaksinen; writing – review & editing, supervision, funding acquisition, conceptualization, resources.

## Conflicts of interest

There are no conflicts to declare.

## Supplementary Material

RA-013-D3RA02807K-s001
