# Supplementary material for: Radiolabelling of peptides with tetrazine ligation based on the inverse electron-demand Diels–Alder reaction: rapid, catalyst-free and mild conversion of 1,4-dihydropyridazines to pyridazines
Source: RSC Adv. 2023 Jul 26;13(32):22606–15. doi: 10.1039/d3ra02807k (PMC10369045; doi:10.1039/d3ra02807k)
Supplement: RA-013-D3RA02807K-s001 [file RA-013-D3RA02807K-s001.pdf]

Electronic Supplementary Material (ESI) for RSC Advances.
This journal is © The Royal Society of Chemistry 2023

SUPPORTING INFORMATION

Radiolabelling of Peptides with Tetrazine Ligation Based on the Inverse Electron-Demand Diels–Alder Reaction: Rapid, Catalyst-free and Mild Conversion of 1,4-Dihydropyridazines to Pyridazines

Sofia Otaru,[a] Tatu Martinmäki,[a] Iida Kuurne,[a] Andreas Paulus,[a] Kerttuli Helariutta,[a]** Mirkka Sarparanta,[a] Anu J. Airaksinen,*[abc]

[a.] *Department of Chemistry, Radiochemistry, University of Helsinki, Finland*
[b.] *Turku PET Centre, University of Turku, Finland*
[c.] *Department of Chemistry, University of Turku*

*anu.airaksinen@utu.fi; Turku PET Centre, University of Turku, Kiinamyllynkatu 4-8, FI-20520 Turku
** Current address: Department of Physics, University of Helsinki, Finland

*Corresponding author: Anu Airaksinen

E-mail: anu.airaksinen@utu.fi

Present address: Turku PET Centre, University of Turku, Kiinamyllynkatu 4-8, FI-20520 Turku

Chemistry

Synthesis of $AmBF_3$-alkyne (**5**) based on a publication by Liu *et al.*[1]

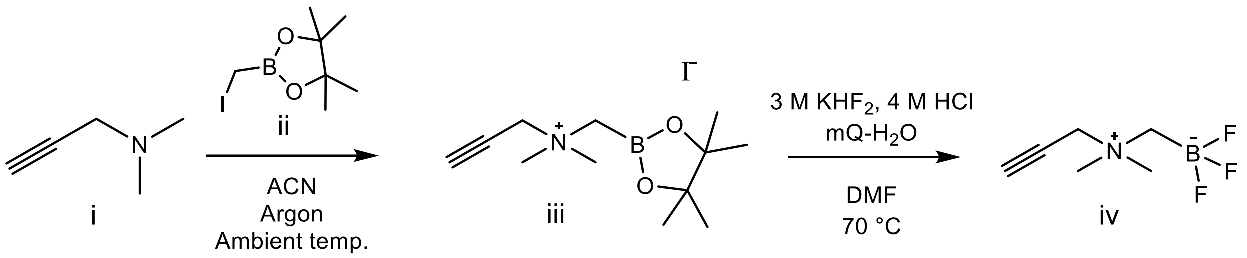

Scheme S1. Synthesis of $AmBF_3$-alkyne (**iv**).

N,*N*-dimethyl-N-[(4,4,5,5-tetramethyl-1,3,2-dioxaborolan-2-yl)methyl]prop-2-yn-1-aminium (**iii**)

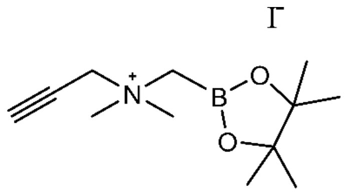

200 µl (186 nmol) of *N*,*N*-dimethylpropargylamine (**i**) was dissolved in 2 mL of anhydrous diethyl ether. 230 µl (126 nmol) of iodoboron pinacol ester (**ii**) in 3 mL of anhydrous diethyl ether was added to the reaction mixture dropwise. After 1.5 hours a light, yellow precipitate formed. The precipitate was filtered and washed with cold diethyl ether until the color turned white. Product **iii** was dried in vacuum for 2 hours. ^{1}H NMR (400 MHz, d_3-$CDCl_3$): δ= 1.32 ppm (s, 12 H), δ= 3.61 ppm (s, 6 H), δ= 4.89 ppm (d, 2 H) ja 2.88 ppm (t, 1 H). ^{13}C NMR (101 MHz, d_3-$CDCl_3$); δ= 86.80 ppm, δ= 82.67 ppm, δ= 72.22 ppm, δ= 60.00 ppm, δ= 57.80 ppm ja δ= 54.07 ppm. A characteristic signal of the pinacol ester moiety was detected the ^{11}B NMR (128 MHz, CD_3CN) at δ= 30.24 ppm.

[Dimethyl(prop-2-yn-1-yl)ammonio]methyltrifluoroborate (**iv**)

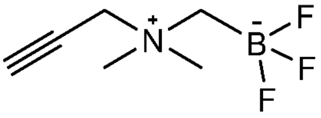

Compound **iii** (50.7 mg) was added into 15 mL polypropylene tube with 200 µl of ultrapure water, 600 µl of dimethylformamide, 300 µl of 3 M potassiumbifluoride (KHF_2) and 300 µl of 4 M hydrochloric acid (HCl). The reaction mixture was heated at 74 °C for 2 hours. Reaction was monitored with TLC (EtOAc:MeOH, 9:1, silica gel plate) with predetermined time-points (t= 30 minutes, 1 h, 2 h). The reaction was quenched by adding 10 µl concentrated ammonium hydroxide (NH_4OH). The final product was purified with SPE cartridges as follows; two Alumina N ja Silica cartridges were preconditioned with 80 mL of water and with 10 mL of solvent mixture (EtOAc:MeOH, 95:5). Reaction mixture was diluted with 6 mL of EtOAc:MeOH (95:5) and applied through the SPE cartridge assembly (2 × silica, 2 × alumina) and the effluent was collected into waste. Compound 4 was eluted out with EtOAc:MeOH (95:5) in 2 mL fractions and analyzed on silica gel TLC. The combined fractions were evaporated with rotary evaporator, and DMF removed with Biotage V-10 Evaporator (Program: Very High Volatile Solvents) in three rounds. The resulting product **iv** was isolated as a white powder in 26.7 mg (72 %) yield. $AMBF_3$-alkyne (**iv**) was characterized with ^{13}C, ^{11}B, ^{19}F and ^{1}H NMR. ^{1}H NMR (400 MHz, CD_3CN): δ= 4.10 ppm (d, 2 H), δ= 3.09 ppm (m, 7 H) and δ= 2.46 ppm (b, 2 H). ^{13}C NMR (101 MHz, CD_3CN), δ= 80.96 ppm, δ= 73.51 ppm, δ=57.72 ppm, δ= 53.42 ppm and δ= 25.10 ppm. Trifluoroborate moiety gives a multiplet signal at δ= -138.87 ppm in ^{19}F NMR (376 MHz, CD_3CN) due to the coupling to boron-11 nucleus. The coupling of ^{11}B with three fluorine atoms gives a quartet signal at δ= 2.22 ppm in ^{11}B NMR spectrum (128 MHz, CD_3CN). In mass spectrometry analysis by TOF-ESI-MS, the following adduct ions were found: 353.1815 $[M+Na]^+$ (calculated m/z=352.9216), 369.1595 $[2M+K]^+$ (calculated m/z=368.8956) and $[2M-F]^+$ 311.1935 (calculated m/z = 310.9216).

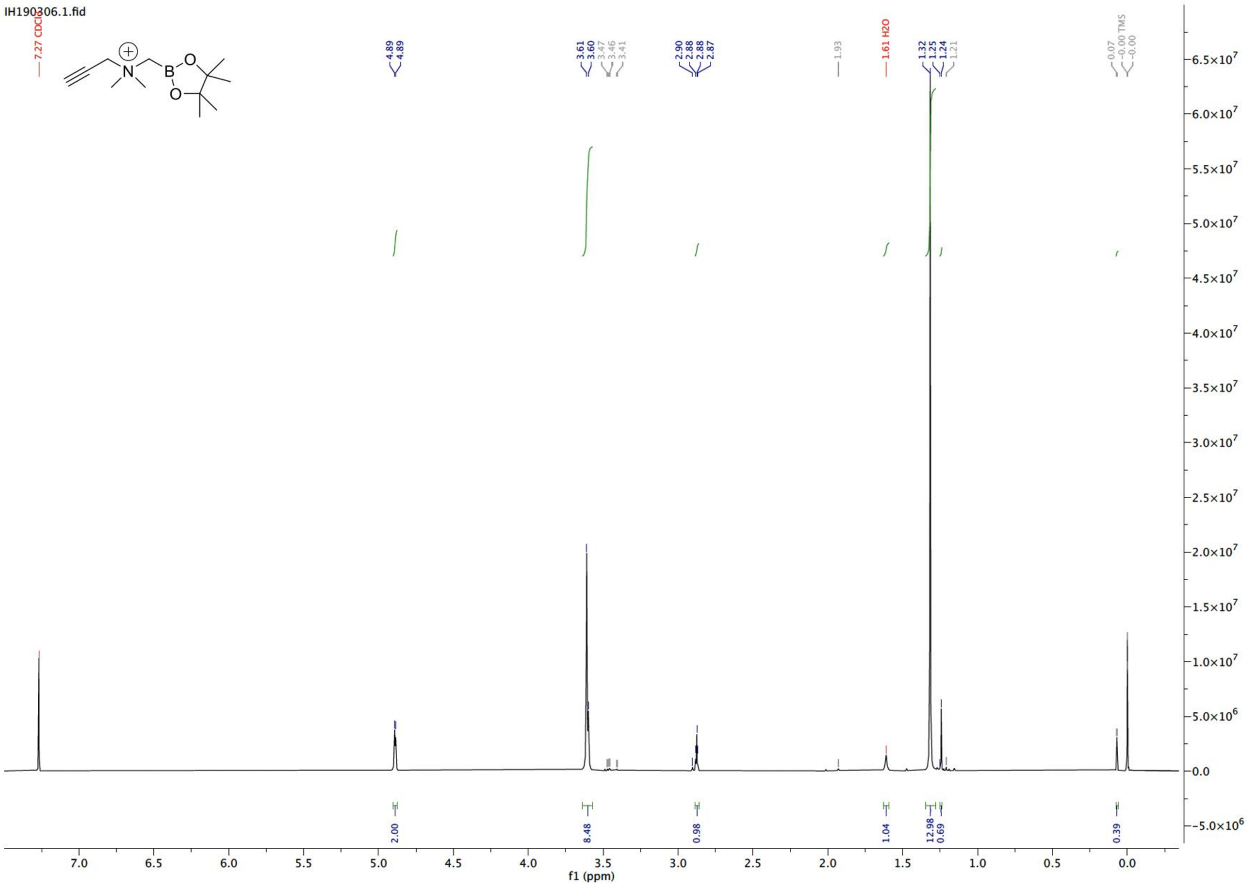

Figure S1. ^{1}H NMR of crude Am-BPin-alkyne (**iii**).

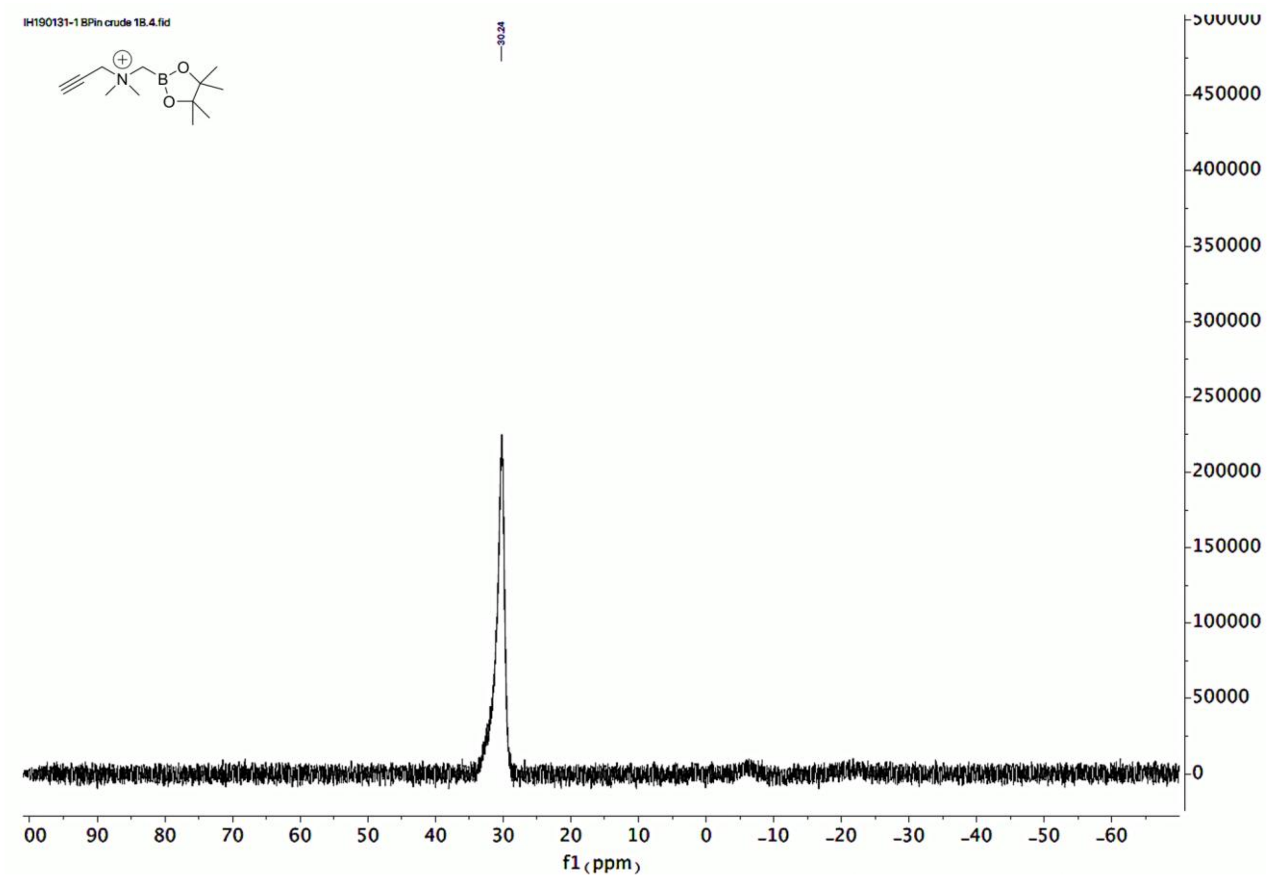

Figure S2. ^{11}B NMR of crude Am-BPin-alkyne (**iii**).

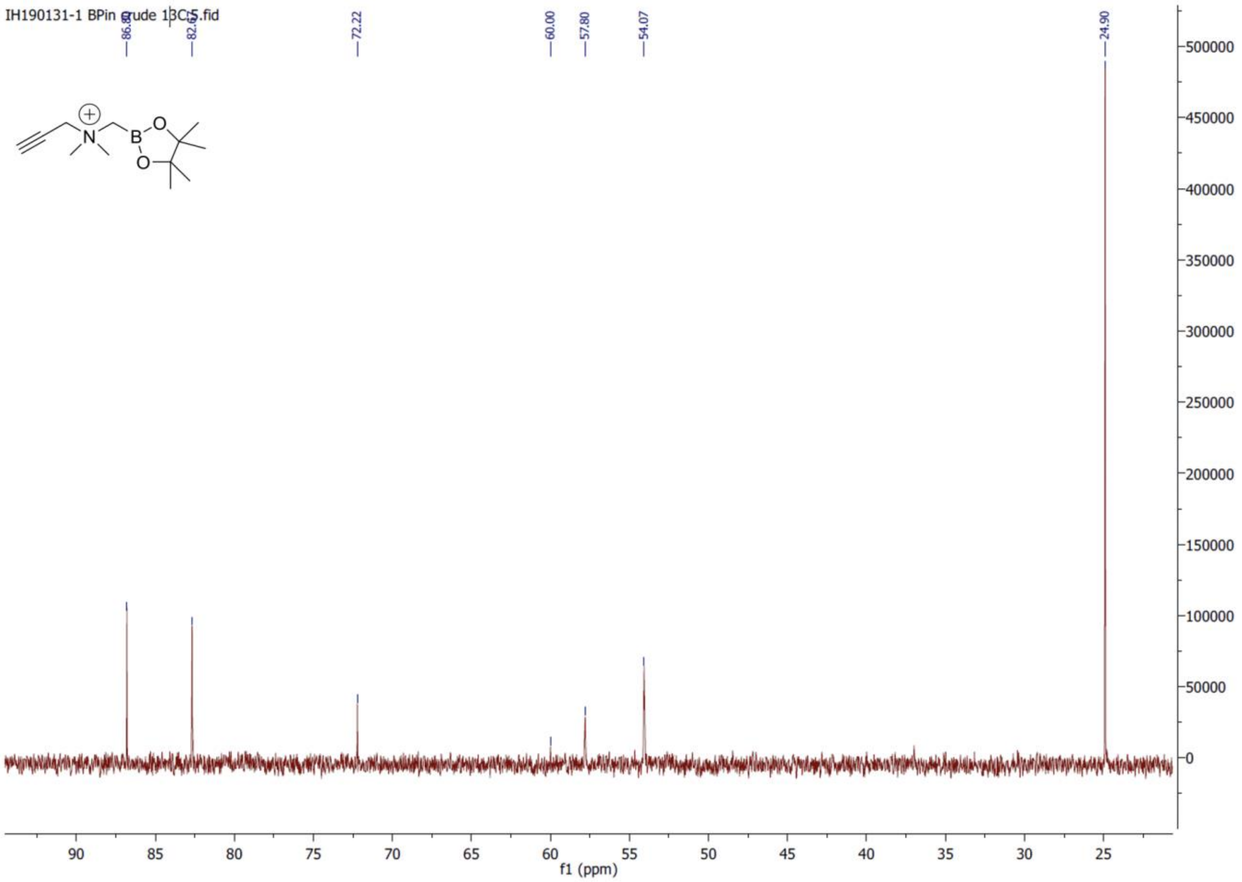

Figure S3. ^{13}C NMR of crude Am-BPin-alkyne (**iii**).

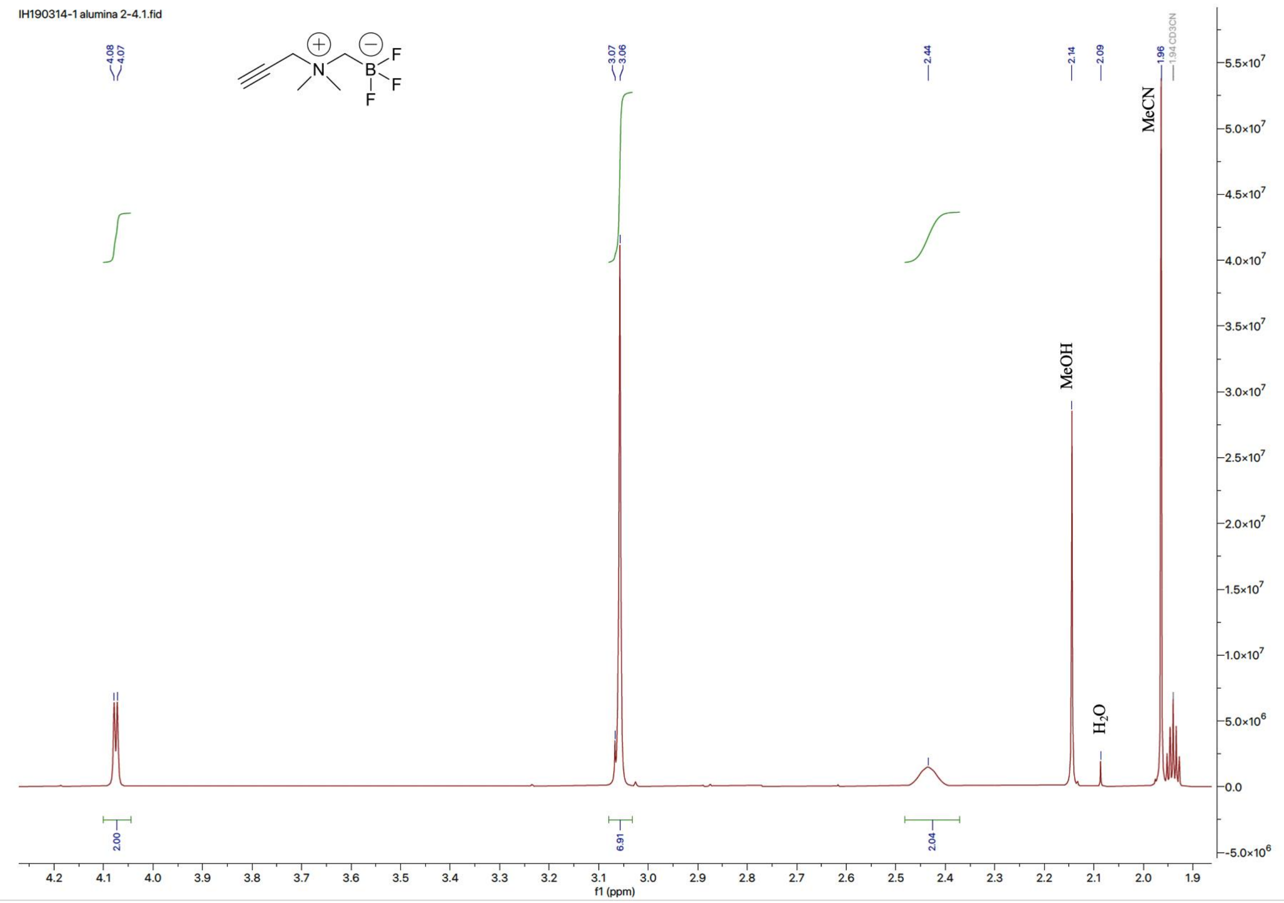

Figure S4. ^{1}H NMR of AmBF$_3$-alkyne (**iv**).

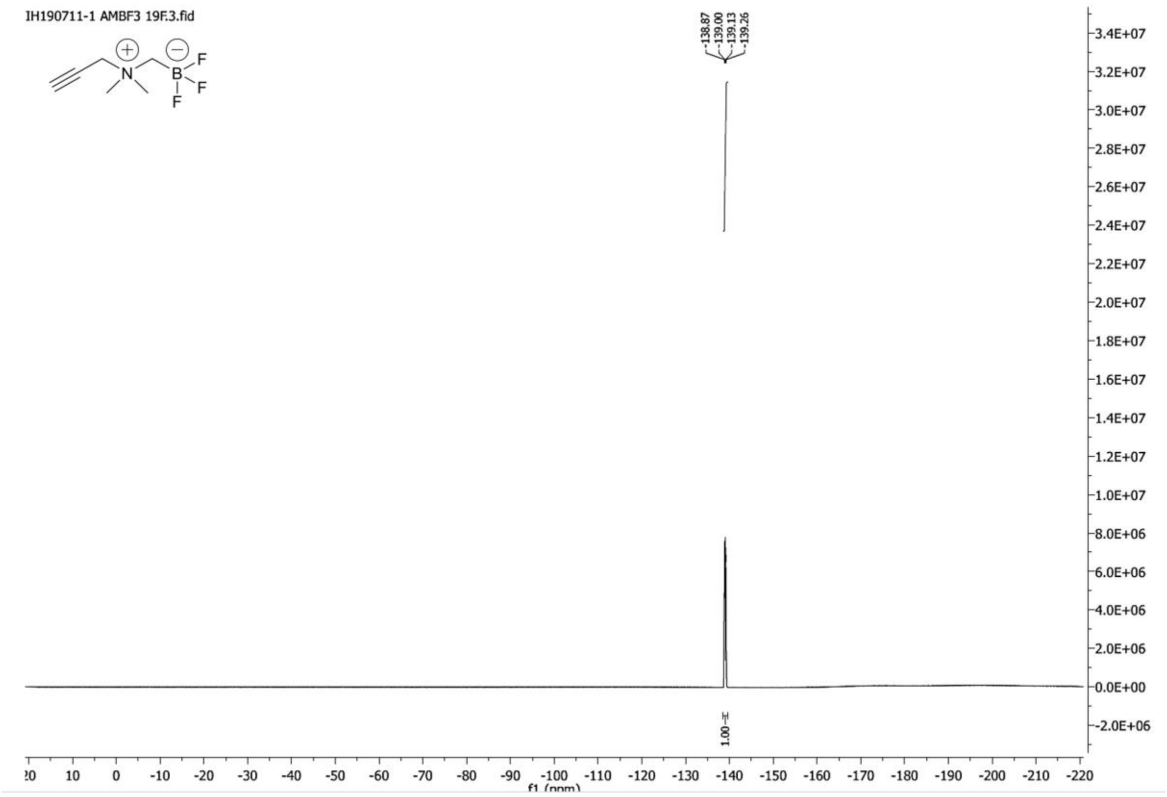

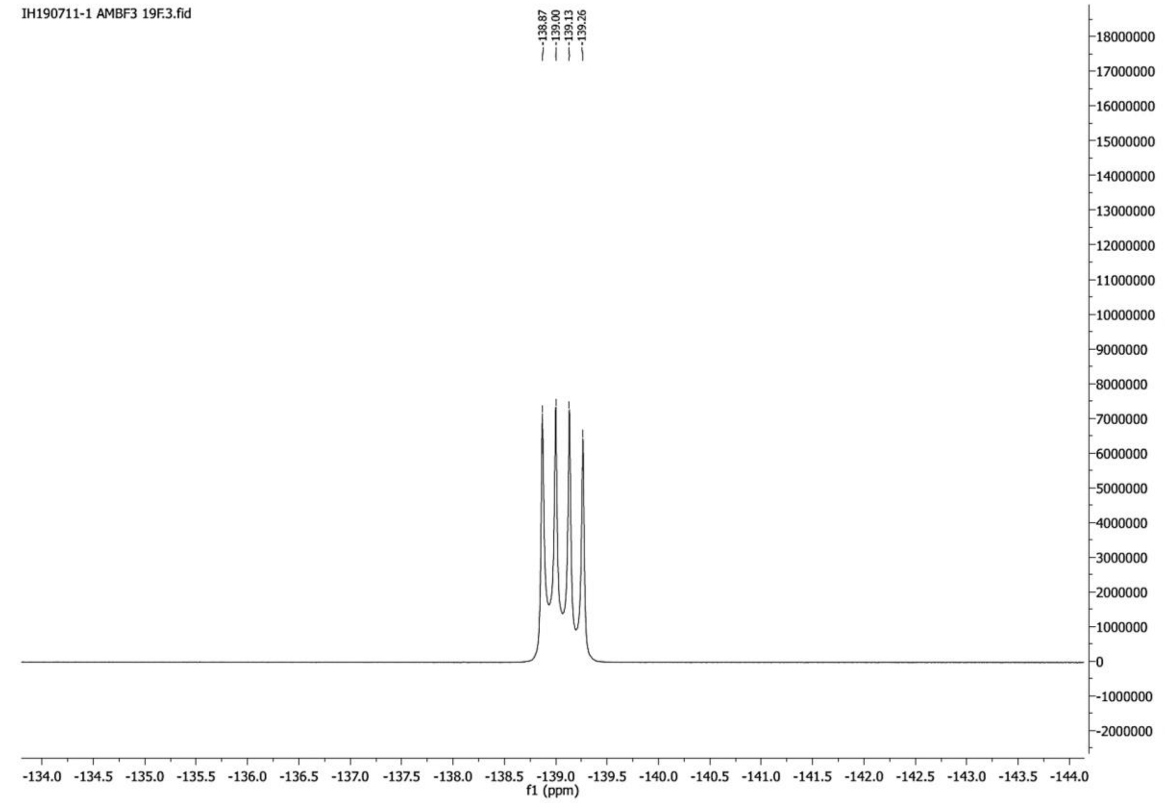

Figure S5. ^{19}F NMR of $AmBF_3$-alkyne (**iv**).

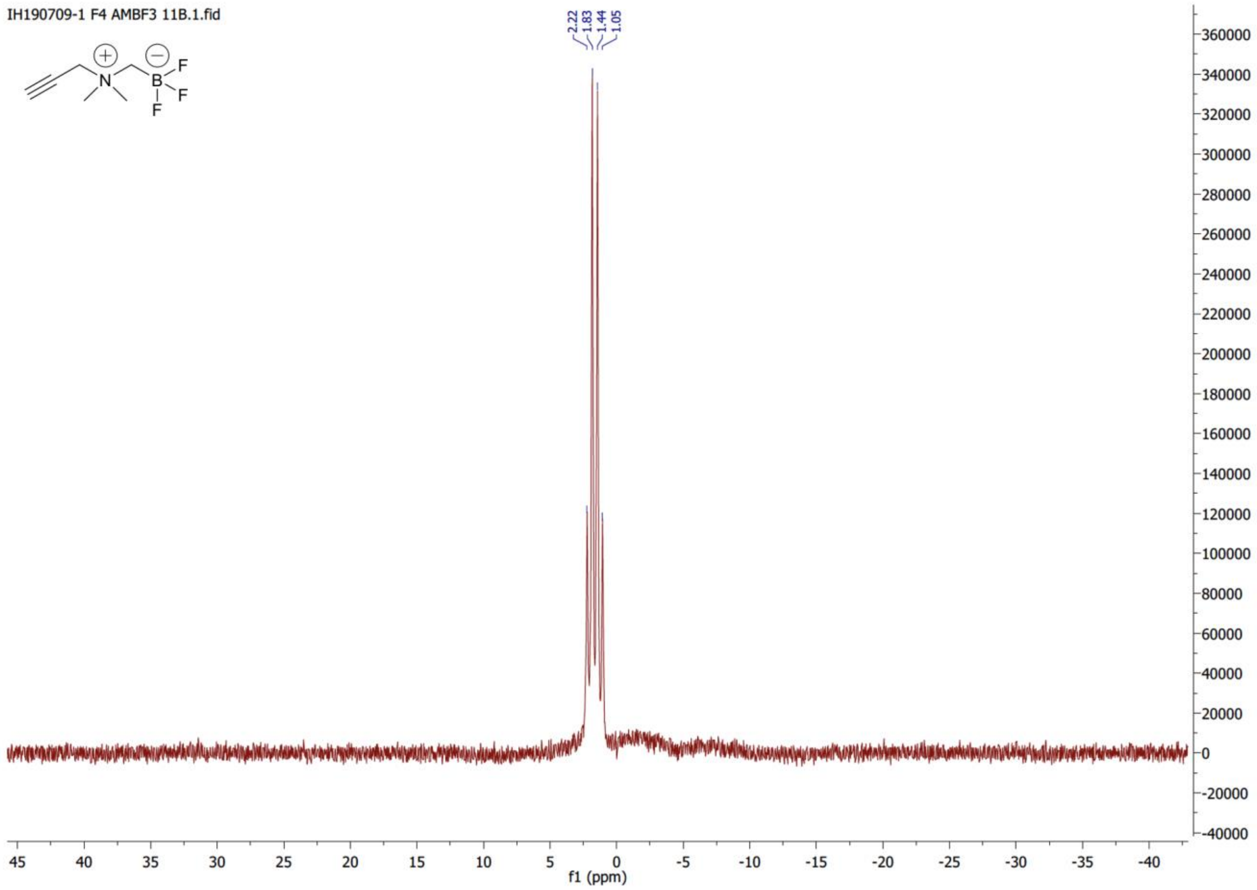

Figure S6. ^{11}B NMR of $AmBF_3$-alkyne (**iv**).

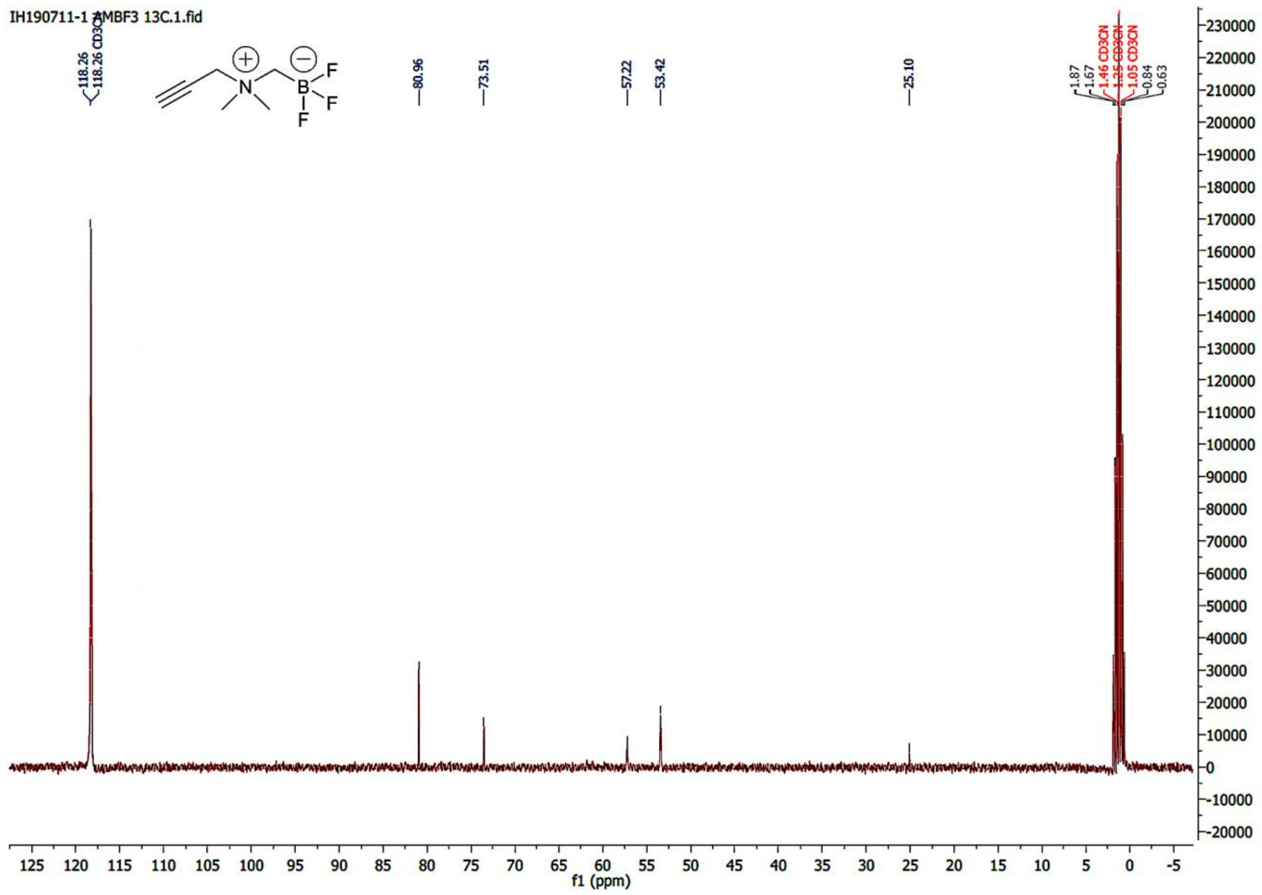

Figure S7. ^{13}C NMR of $AmBF_3$-alkyne (**iv**).

Synthesis of $AmBF_3$-PEG_4-mTz (**vi**)

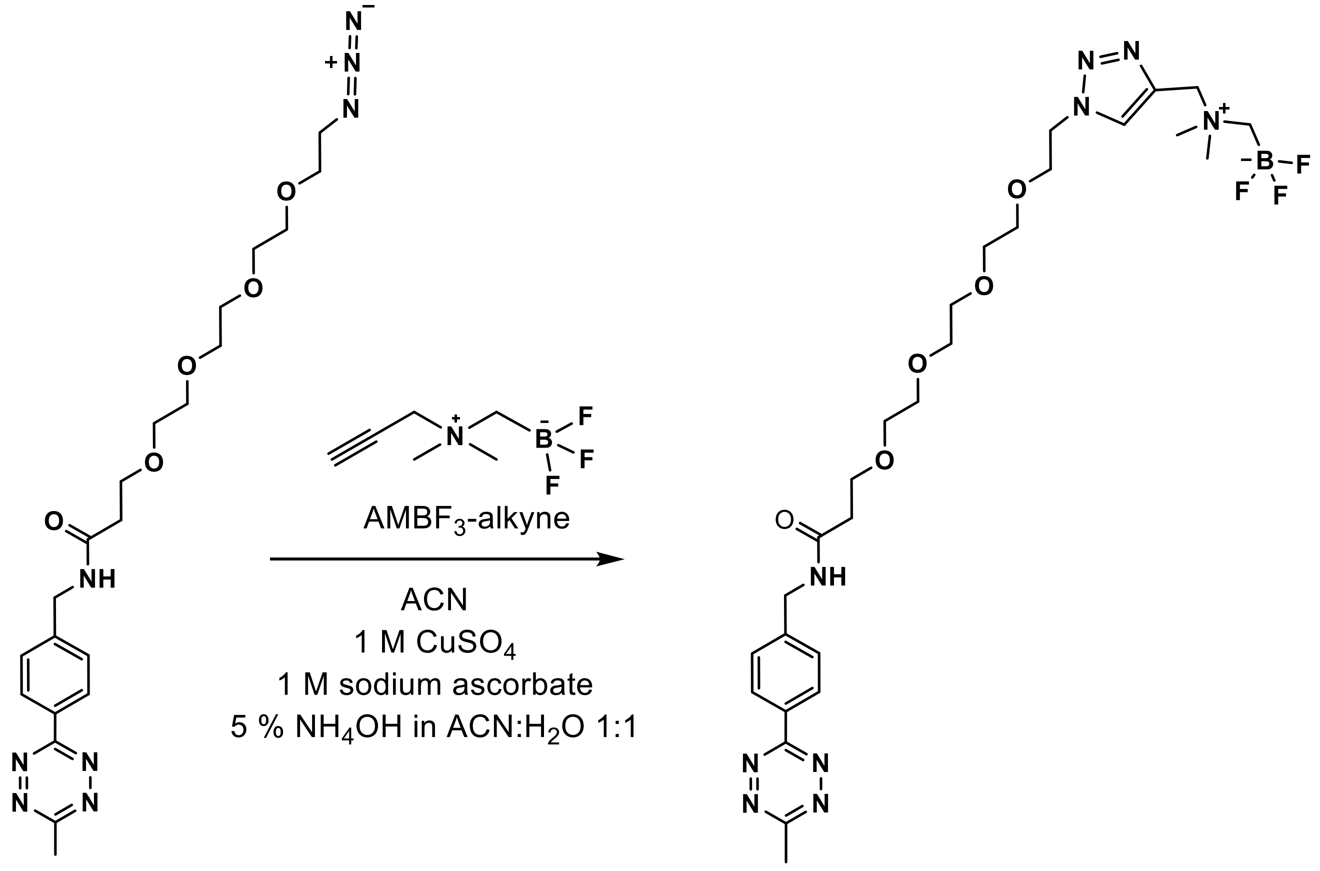

v **vi**

Scheme S2. Synthesis of $AmBF_3$-PEG_4-mTz (**vi**).

({[(1-{1-[4-(1,2,4,5-tetrazin-3-yl)phenyl]-3-oxo-6,9,12,15-tetraoxa-2-azaheptadecan-17-yl}-1H-1,2,3-triazol-4-yl)methyl]dimethylammonio}methyl)trifluoroborate (**vi**).

4.0 mg (8.5 µmol) mTz-PEG_4-azide (**v**) in 87 µL of ACN (DNA synthesis quality), 10 µL of 1 M $CuSO_4$, 25 µL of 1 M sodium ascorbate, 100 µL of 5 % NH_4OH in ACN:H_2O 1:1, 100 µL of H_2O and 4.5 mg (27.3 µmol) of $AMBF_3$-alkyne in 80 µL of ACN were mixed and incubated at 45 °C for 150 minutes with stirring. The product (**vi**) was purified with Sep-Pak C18 Plus cartridge. ^1H NMR (400 MHz, *CD_3CN*): δ 8.47 (d, J = 8.4 Hz, 2H): 8.10 (s, 1H): 7.53 (d, J = 8.4 Hz, 2H): 7.16 (s, 1H): 4.58 – 4.50 (m, 2H): 4.49 – 4.43 (m, 4H): 3.83 (dd, J = 5.6, 4.7 Hz, 2H): 3.71 (t, J = 6.0 Hz, 2H): 3.52 (m): 2.98 (d, J = 21.1 Hz, 10H): 2.45 (t, J = 6.0 Hz, 2H): 2.15 (s, 3H). ^{13}C NMR (101 MHz, *CD_3CN*): δ 172.28, 38: 168.61: 164.98: 145.68: 137.51: 132.11: 130.48 – 127.52 (m): 71.22-69.98 (m): 68.10: 61.63: 53.67: 51.32: 43.44: 37.70: 21.56. Detected $[M-F]^+$=620.5 m/z, calculated $[M-F]^+$=620.3 m/z.

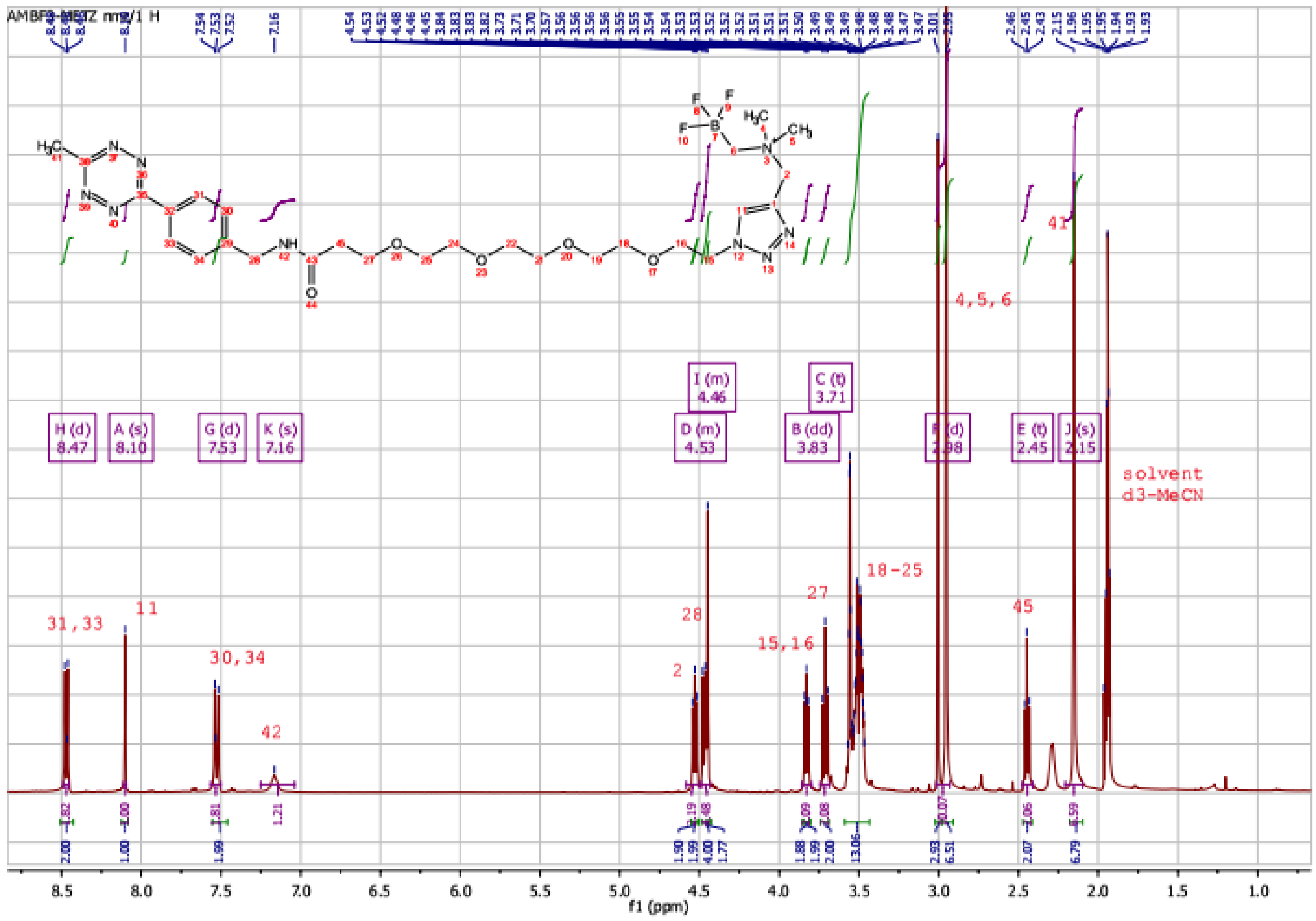

Figure S8. ^{1}H NMR of $AMBF_3$-PEG_4-mTz (**vi**).

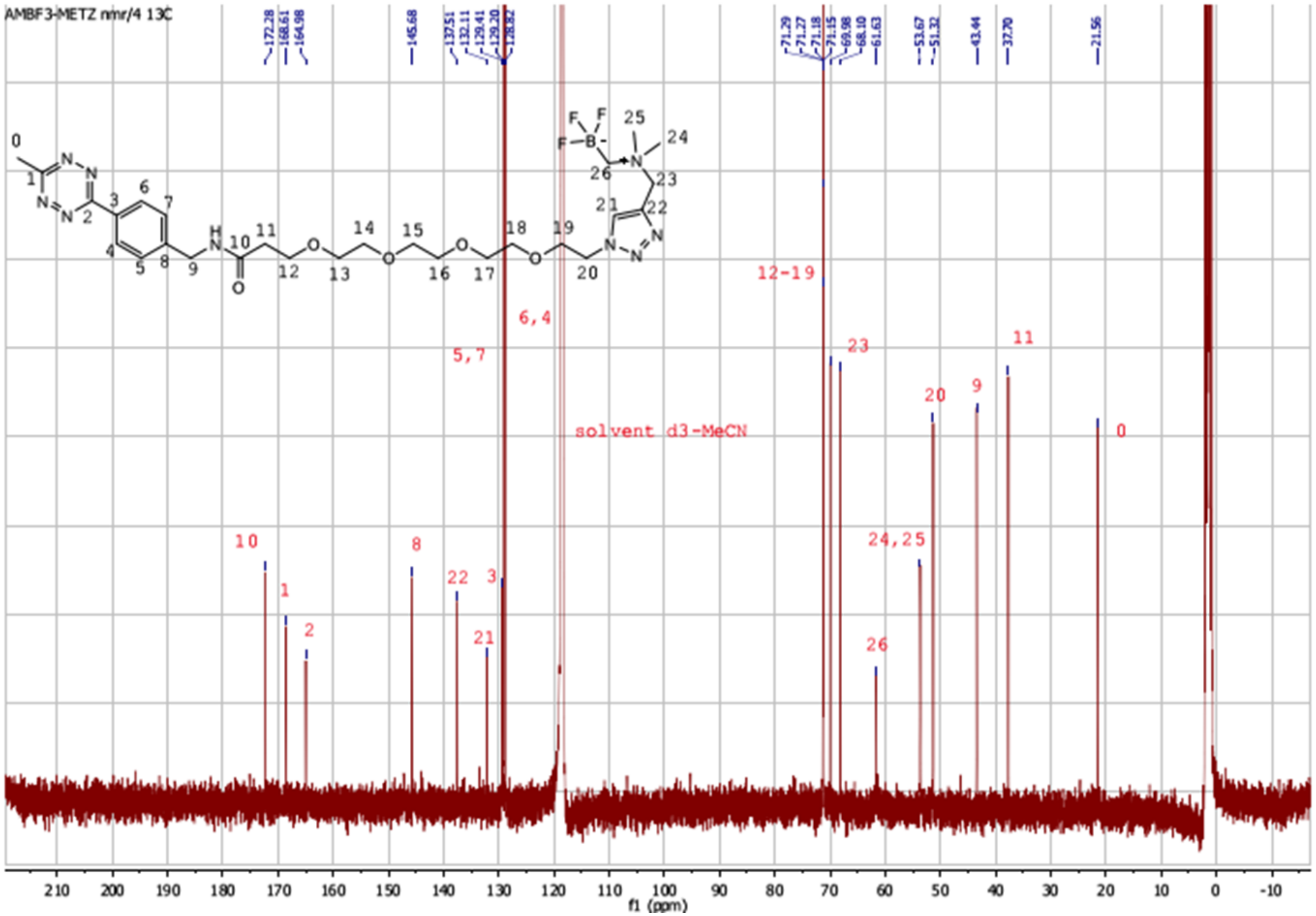

Figure S9. ${}^{13}C$ NMR of $AMBF_3$-PEG_4-mTz (**vi**).

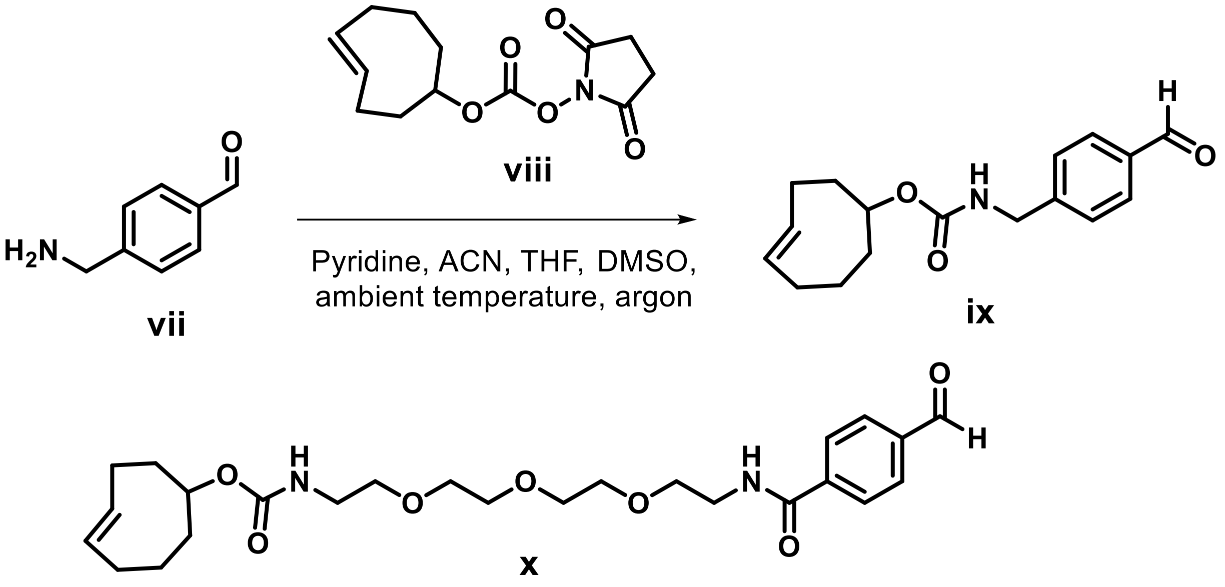

Figure S10. Synthesis of TCO-CHO (**ix**),[2] and chemical structures of TCO-aldehydes TCO-CHO (**vii**) and TCO-PEG3-CHO (**x**) used for the functionalization of amino-oxy peptides.

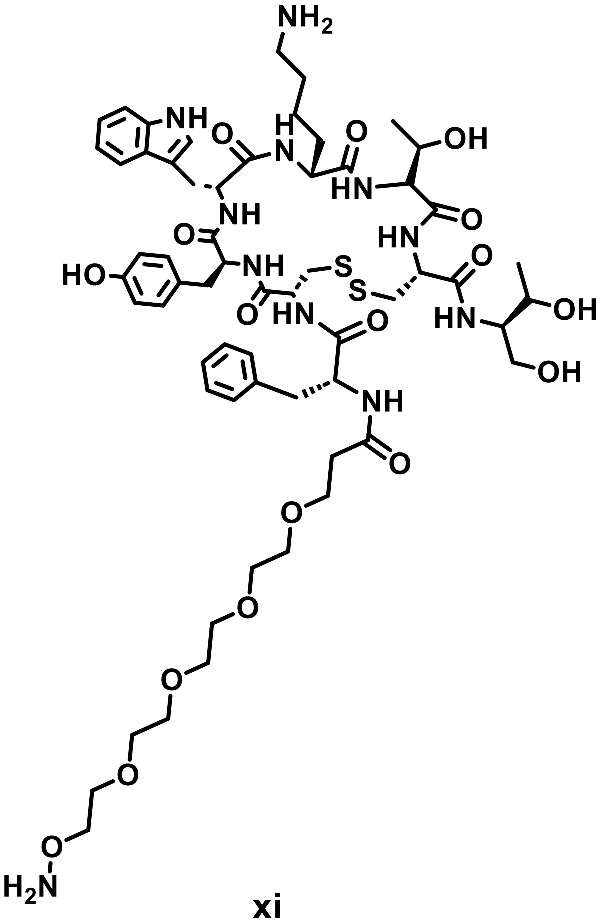

Figure S11. Chemical structures of peptide derivative TOC-PEG$_4$-ONH$_2$ (**xi**).

UHPLC-HRMS

Selected compounds were analysed by UHPLC Thermo Scientific Dionex Ultimate 3000 ultrahigh performance liquid chromatography (Germering, Germany) which was coupled to a Thermo Scientific Orbitrap Fusion mass spectrometer (San Jose, CA, USA). Ionization was executed with a heated electrospray ionization (HESI) source operated in positive ionization mode ($HESI^+$). The scan range was set at 120–1200 *m*/*z* and 100–2000 *m*/*z.* The acquired data was processed with Xcalibur workstation (Thermo Fisher Scientific, Waltham, MA, USA). Selected peptide derivatives were analyzed for the detection of their exact masses and proposed elemental compositions, including mass errors.

Table S1. The gradient used for UHPLC-HRMS studies.

Time (min)	%B
0	5
3	5
4	25
5	60
6	80
7	100
7.5	5
9	5

Flow rate: 0.5 mL/min at 40 °C
Column: Waters ACQUITY UPLC® 1.7 µm BEH C18 130Å, UPLC Column 2.1 × 50 mm
Eluents: (A) H_2O+0.1% Formic acid and (B) ACN+0.1% Formic acid

Table S2. The gradient used for HPLC-RAD/DAD studies.

Time (min)	%B
0	20
20	50
23	20
25	20
32	20

Flow rate: 3 mL/min at room temperature
Column: Phenomenex Kinetex® 5 µm C18 100 Å, LC Column 250 × 10.0 mm
Eluents: (A) H_2O+0.1% Trifluoroacetic acid and (B) ACN +0.1% Trifluoroacetic acid

LC-MS analysis*:* Selected compounds were analyzed with Agilent Technologies 1260 Infinity HPLC-DAD system with Agilent Technologies 6120 Quadrupole LC/MS detector. Ionization was executed with electrospray ionization in positive mode (ESI^+), at a scan range of scan range 100-2000 *m/z*. Data was processed with OpenLAB CDS Workstation.

Table S3. The gradient used for HPLC-DAD-ESI-MS studies.

Time (min)	%B
0	10
33	90
35	90
40	10

Flow rate: 0.7 mL/min at room temperature
Column: Waters Atlantis® T3 3 µm C18 100 Å, LC Column 4.6 × 150 mm
Eluents: (A) 0.1 % Formic acid in water (B): 0.1 % Formic acid in ACN

Functionalization of peptides with TCO

Amino-oxy peptide TOC-PEG$_4$-ONH$_2$ (**xi**) was purchased from a commercial provider (CS Bio, Menlo Park, CA, USA) as a custom synthesis, and was modified by oxime bond formation with TCO-CHOs. Aminooxy peptide (1.0 eq.) was dissolved in 600 µL of 0.3 M anilinium acetate buffer (pH 4.6) and stirred while adding TCO-aldehyde (**ix** or **x**) (1.5 eq.) dissolved in 20 µL of chloroform. The reaction mixture was stirred for 1–2 hours at room temperature and monitored by HPLC (PDA = 280 nm). The resulting TCO-peptides were purified with HPLC, and the collected fraction immediately used as such for radiolabeling with [^{18}F]AmBF$_3$-Tz or [^{18}F]AmBF$_3$-PEG$_4$-mTz. The crude reaction mixtures were analyzed with HPLC.

Table S4. The retention times (HPLC-RAD/DAD and HPLC-RAD/UV methods), Log$D_{7.4}$ values and purity percentages (%) of dihydropyridazine (DHP) and pyridazine forms of the peptides, after HPLC separation.

Radiotracer		LogD7.4	Retention time (min)	Ratio (%)
[^{18}F]1	[^{18}F]AmBF$_3$-Tz	-0.13 ± 0.06 (n=4)	11.6	100
[^{18}F]5 (ox.)	[^{18}F]AmBF$_3$-PEG$_4$-TOC	0.58 ± 0.06 (n=4)	18.7	100
[^{18}F]6 (ox.)	[^{18}F]AmBF$_3$-PEG$_7$-TOC	-0.73 ± 0.12 (n=4)	17.3	100
[^{18}F]6 (DHP, a, b cluster)	[^{18}F]AmBF$_3$-PEG$_7$-TOC	-0.04 ± 0.02 (n=3)	20.2–20.6	100
[^{18}F]5 (ox.)	[^{18}F]AmBF$_3$-PEG$_4$-TOC	0.58 ± 0.06 (n=4)	18.7	100
[^{18}F]6 (DHP, a)	[^{18}F]AmBF$_3$-PEG$_7$-TOC	-0.21 ± 0.19 (n=3)	20.6	77
[^{18}F]6 (DHP, b)	[^{18}F]AmBF$_3$-PEG$_7$-TOC	0.28 ± 0.16 (n=3)	20.2	88
[^{18}F]7 (ox.)	[^{18}F]AmBF$_3$-PEG$_{11}$-mTOC	not analyzed	17.3	100
[^{18}F]7 (DHP cluster)	[^{18}F]AmBF$_3$-PEG$_{11}$-mTOC	not analyzed	20.6–21.0	[1*]

[1*] The cluster of peaks together without separating the different analogs

Table S5. Retention times of peptide derivatives

**Compound #**	**Name**	**Retention time t_R (min)**	**Analysis method**
**3***	TCO-PEG$_4$-TOC	22.3	HPLC-DAD
**4***	TCO-PEG$_7$-TOC	24.1	HPLC-DAD
[^{18}F]**1**	[^{18}F]AmBF$_3$-Tz	10.3	HPLC-DAD/ Radio-HPLC
[^{18}F]**2**	[^{18}F]AmBF$_3$-PEG$_4$-mTz	14.0	HPLC-DAD/ Radio-HPLC
[^{18}F]**5**	[^{18}F]AmBF$_3$-PEG$_4$-TOC		
	Oxidized	18.5	HPLC-DAD/ Radio-HPLC
[^{18}F]**6**	[^{18}F]AmBF$_3$-PEG$_7$-TOC		
	Oxidized	17.2	HPLC-DAD/ Radio-HPLC
	Reduced	17.6-18.5	HPLC-DAD/ Radio-HPLC
	Oxidized	11.6	HPLC-DAD-ESI
	Reduced	12.7	HPLC-DAD-ESI
	Oxidized	4.93	UHPLC-HRMS
	Reduced		UHPLC-HRMS
[^{18}F]**7**	[^{18}F]AmBF$_3$-PEG$_{11}$-mTOC		
	Oxidized	18.6	HPLC-DAD/ Radio-HPLC
	Reduced	20.7	HPLC-DAD/ Radio-HPLC
	Oxidized	4.81	UHPLC-HRMS
	Reduced	5.05-5.07	UHPLC-HRMS

*Compounds published earlier by our group.[2]

Table S6. Pyridazine HCl buffer recipe used for the radiosynthesis of [^{18}F]AmBF$_3$-tetrazines.[1]

**Component**	**Volume (µL)**
Pyridazine	360
ACN	3160
DMF	660
water	590
37% HCl	230
V_{tot}	5000

Lipophilicity. Shake-flask method was used for determining the lipophilicities (Log$D_{7.4}$) of the radiolabeled compounds. Log$D_{7.4}$ was determined as a distribution of radioactivity between 0.01 M PBS and octanol. The purified radiolabeled compound (25 µL) was added to a 1:1 mixture of 1-octanol and 0.02 M PBS (pH 7.4) in a 1.5 mL microtube. The mixture was shaken mechanically at 500 rpm for 10 minutes, centrifuged (1000 × *g*, 5 minutes), and the layers were separated, followed by pipetting a sample (500 µL) from each layer into a pre-weighed polypropylene tube. The radioactivity in the sample was measured with a Wizard gamma counter.

The distribution of the radiolabeled compound between the octanol and the PBS layers was calculated according to equation:

$$LogD_{7.4} = Log\frac{A_{Octanol}}{A_{PBS}}$$

A_{oct}=activity in the octanol phase

A_{PBS}=activity in the PBS phase

Cell-uptake assay in AR42J

AR42J by ATCC®, Virginia, USA (CRL-1492™) cells were used for *in vitro* biological evaluation. T175 flasks were used, the cells were cultured in 37 °C with 5 % CO_2. The cells were cultured in Gibco™ F-12K Medium by Thermo Fischer Scientific, Waltham, USA with 20 % FBS (Gibco™) and 1 % PenStrep by Sigma-Aldrich, Saint Louis, USA. CO_2-independent medium (Gibco™) with 20 % FBS, 1 % PenStrep and 1×GlutaMax (Gibco™) was used as a growth medium during the cell studies. 0.05 M Glycine buffer pH 2.8 was prepared by dissolving 1.40 g glycine hydrochloride (Sigma Aldrich) to 230 mL of water, adjusting the pH to 2.8 with 0.1 M NaOH and filling with water to final volume of 250 mL. 1×PBS was prepared from Phosphate Buffered Saline Tablets (Fisher Bioreagents, Hampton, USA) in water. 1.0 M Sodium hydroxide was purchased from VWR Chemicals. Cell farctions were collected to Perkin Elmer 6 mL PE scintillation tubes. AR42J cells (1×10^6 per well) were seeded overnight on 6-well plates. The cell growth media was removed and the reaction media containing tracer was added and the cells were incubated at 37 °C. Another set of cells were co-incubated in the presence of 1 µM solution of non-modified octreotide for studying the specificity of the cell-uptake. At designated time-points (15, 30, 60 and 120 minutes) the reaction media was removed and collected to a microtube, followed by washing the cells with 1 mL of cold 1×PBS and collecting the supernatant into the same microtube (=free fraction). The membrane-bound fraction was collected by adding cold glycine buffer (1 mL) onto the cells, by incubating for 5 minutes on ice, removing the supernatant, repeating the procedure, and washing the cells with cold 1×PBS. All of the supernatants were collected to the same microtube. 1 M NaOH was added on the cells and left to incubate in ambient temperature for 10 minutes. The supernatant was removed, the cells were washed twice with cold 1×PBS, and the supernatants were collected into the same microtube (=internalized fraction). The supernatants collected separately in each phase, were measured with a gamma counter for determining the radioactivity % of each fraction.

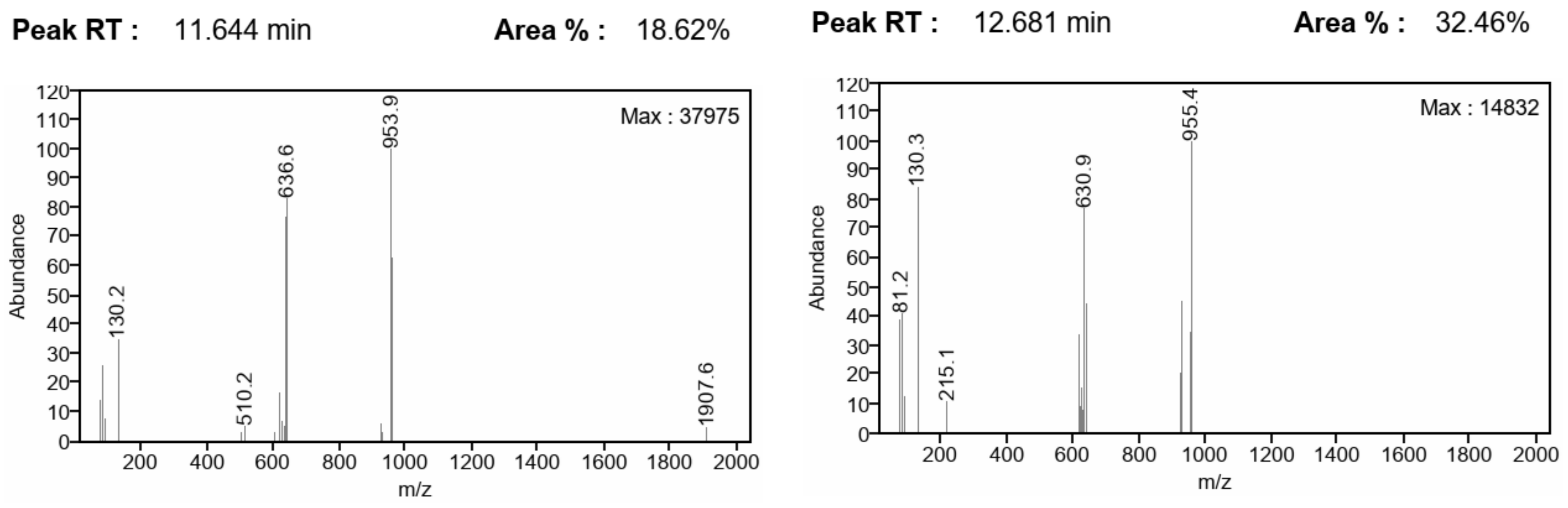

Figure S12. HPLC-DAD-ESI-Mass spectrum of **5** as its oxidized AmBF$_3$-PEG$_4$-TOC (left) $[M+2H]^{2+}$ and reduced AmBF$_3$-PEG$_4$-TOC (right) $[M+2H]^{2+}$.

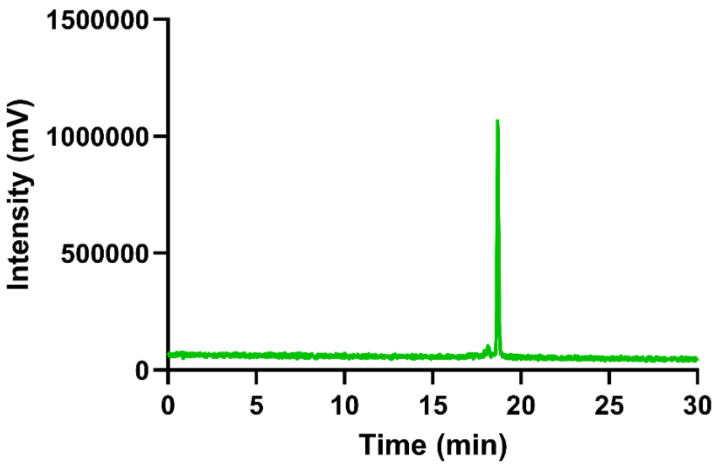

Figure S13. Radio-HPLC chromatogram of [^{18}F]AmBF$_3$-PEG$_4$-mTz ([^{18}F]**7**).
t_R = 18.6 min (mV).

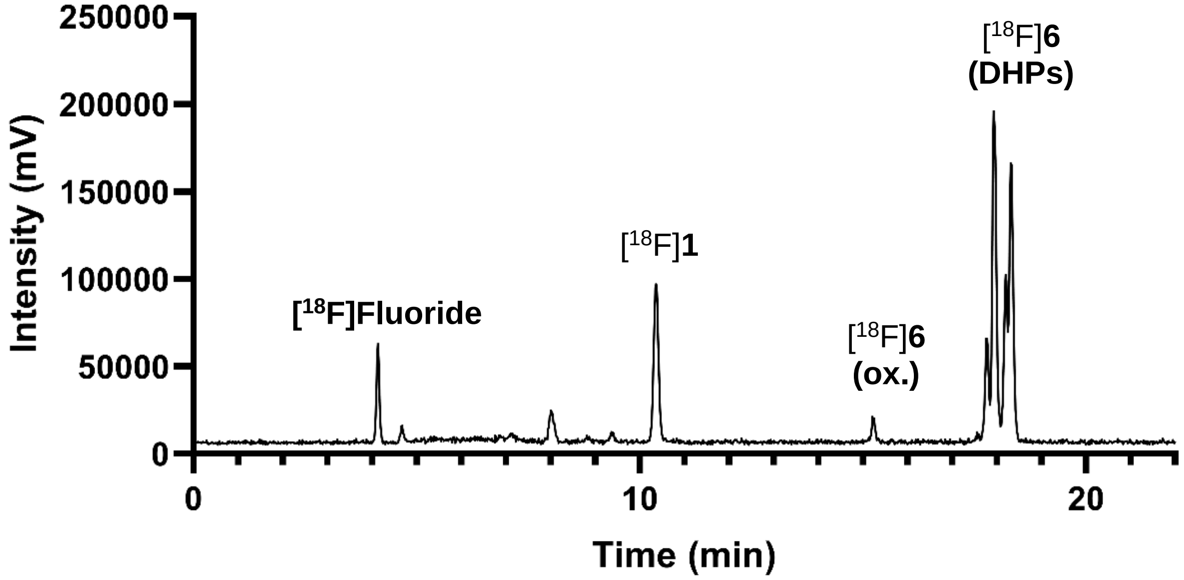

Figure S14. Radio-HPLC chromatogram of crude mixture of [^{18}F]AmBF$_3$-PEG$_7$-TOC ([^{18}F]**6**) demonstrating [^{18}F]**1** at t_R =10.3 min, oxidized [^{18}F]**6** at 15.2 min and reduced [^{18}F]**6** at 17.6-18.5 min. *HPLC conditions were later optimized and retention times were approximately 2 minutes later for radiolabeled TOCs [^{18}F]**6(ox.)** (t_R≈17 min) and [^{18}F]**6(DHPs)** (t_R≈20 min).

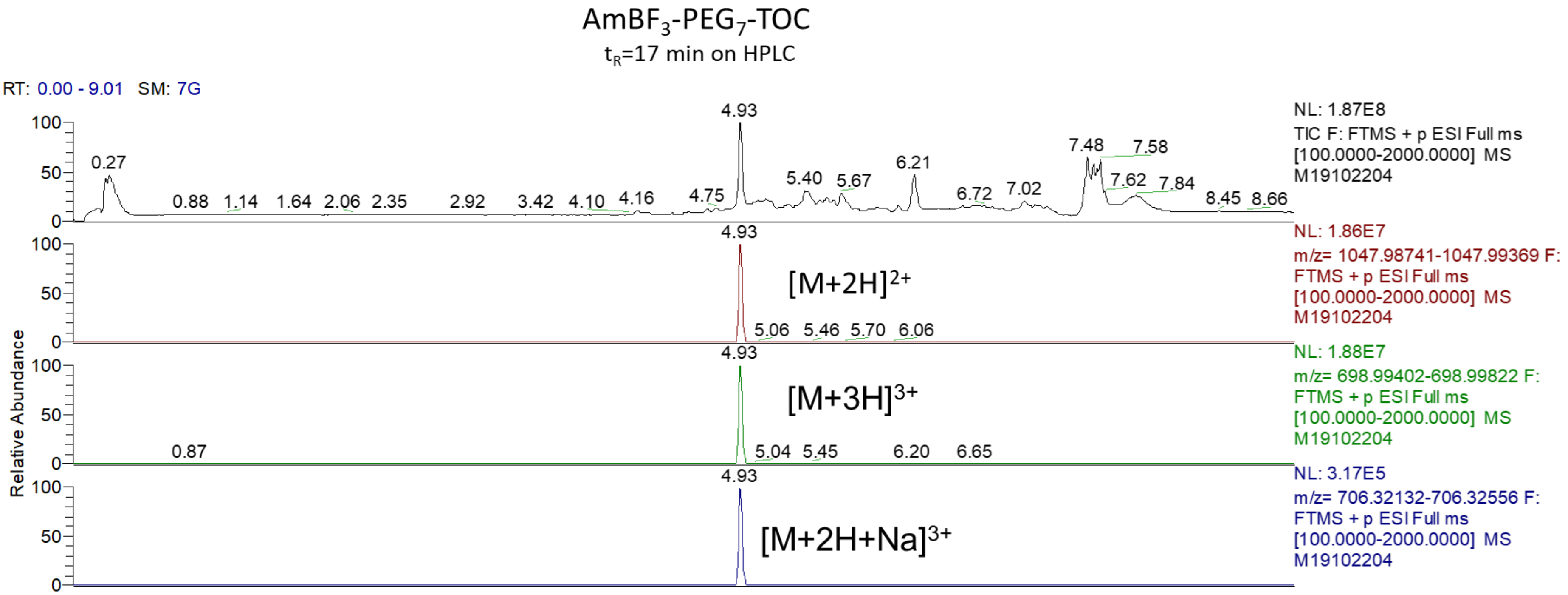

Figure S15. UHPLC-HRMS total ion chromatogram (TIC) together with extracted ion chromatograms (EICs) of crude mixture of $AmBF_3$-PEG_7-TOC (**6**) after 18 hours incubation in HPLC fraction at room temperature demonstrating oxidized form of the peptide at retention time of t_R=4.93 as molecule ion peaks $[M+2H]^{2+}$, $[M+3H]^{3+}$ and $[M+2H+Na]^{3+}$.

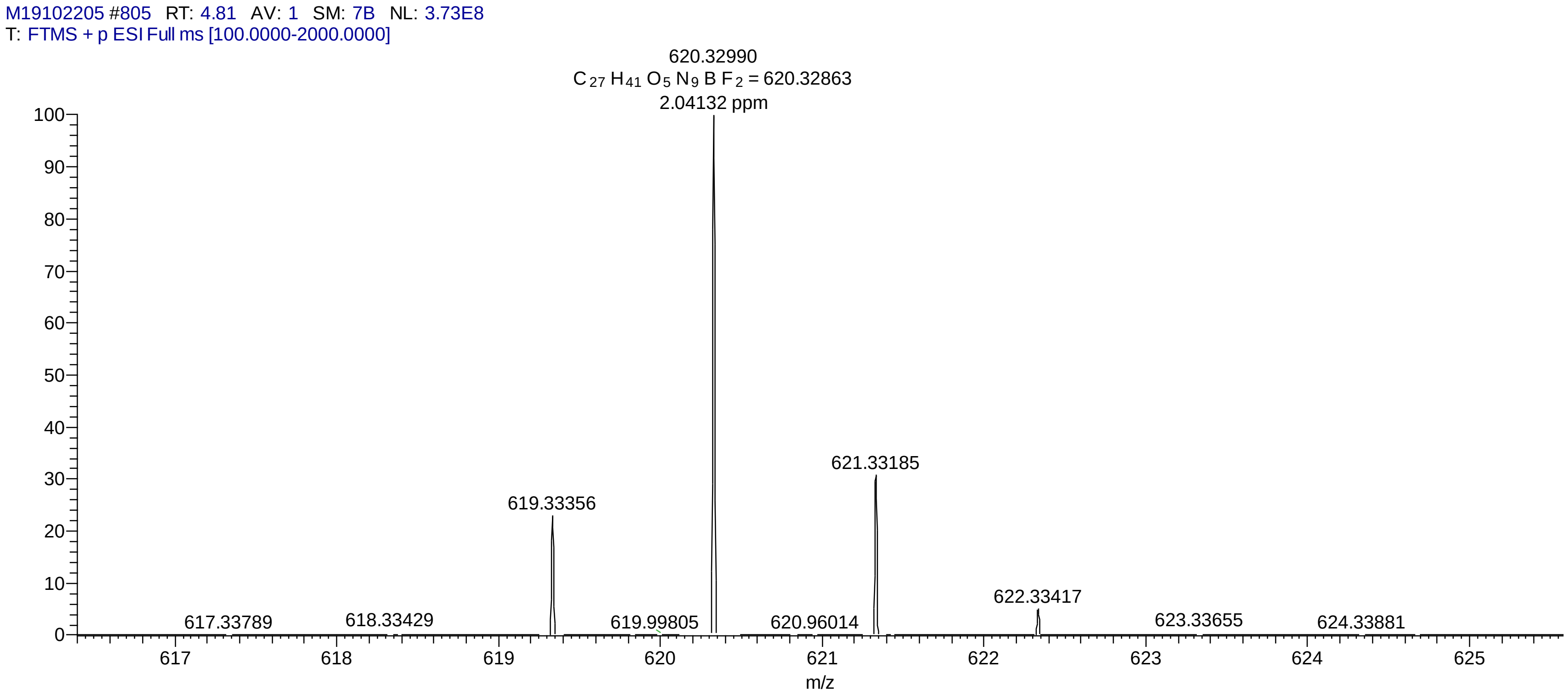

Figure S16. UHPLC-HRMS extracted ion chromatogram of $AmBF_3$-PEG_4-mTz (**2**) with a retention time of t_R=4.81 min, in crude reaction mixture after IEDDA cycloaddition between **2** with **4** as molecule ion peaks $[M+2H]^{2+}$ with mass error of 2.04132 ppm calculated *m/z* 620.32863 for $C_{27}H_{41}O_5N_9BF_2$, meas; *m/z* 620.32990.

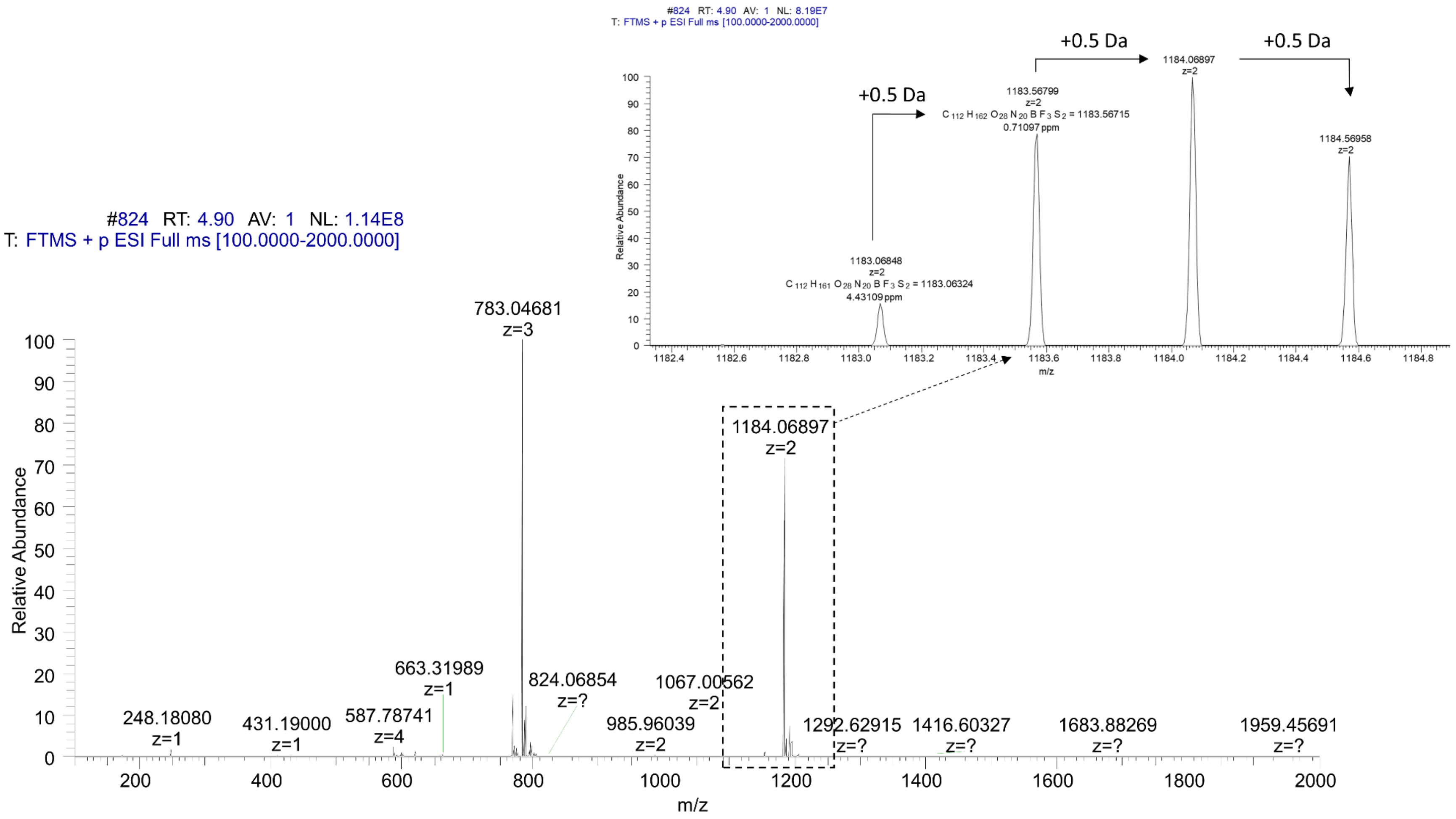

Figure S17. UHPLC-HRMS extracted ion chromatogram of $AmBF_3$-PEG_{11}-mTOC (**7**) oxidized form with a charge of Z=2 and a retention time of t_R=4.90 min in crude reaction mixture after IEDDA cycloaddition between **2** with **4**. Compound **7** was detected as doubly charged $[M+2H]^{2+}$ ion with mass error of Δ=0.71097 ppm (calculated *m/z* 1183.56715 for; $C_{112}H_{162}O_{28}N_{20}BF_3S_2^{2+}$, meas; *m/z* 1183.56799*).* Isotopic peak pattern of 0.5 Da indicates the peptide is detected in charge state +2.

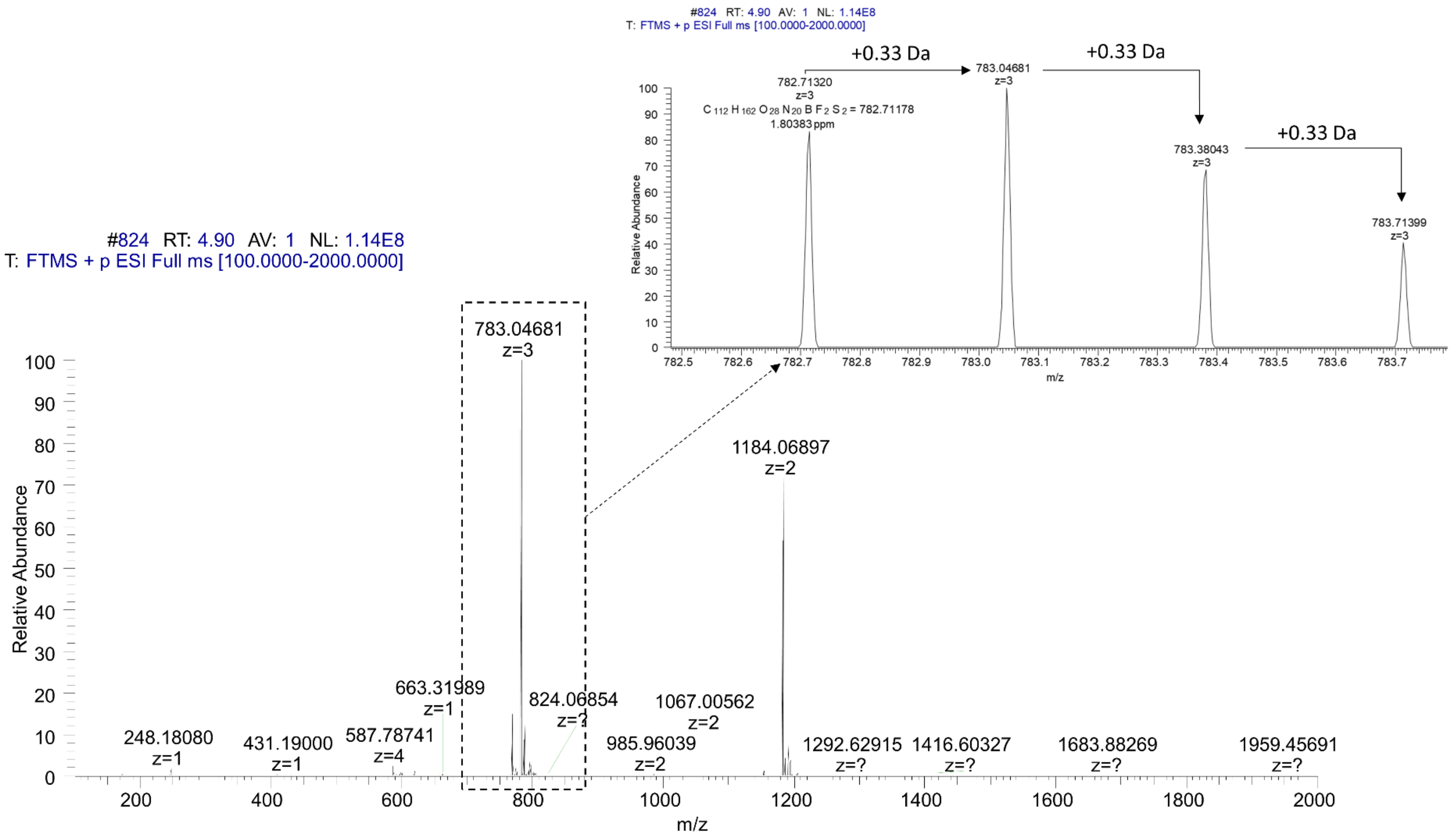

Figure S18. UHPLC-HRMS extracted ion chromatogram of AmBF$_3$-PEG$_{11}$-mTOC (**7**) oxidized form with a charge of Z=3 and a retention time of t_R=4.90 min in crude reaction mixture after IEDDA cycloaddition between **2** with **4**. Compound **7** was detected as triply charged $[M+3H-F]^{3+}$ ion with mass error of Δ=1.80383 ppm (calculated *m/z* 782.71178 for; $C_{112}H_{162}O_{28}N_{20}BF_2S_2^{3+}$, meas; *m/z* 782.71320*).* Isotopic peak pattern of 0.33 Da indicates the peptide is detected in charge state +3.

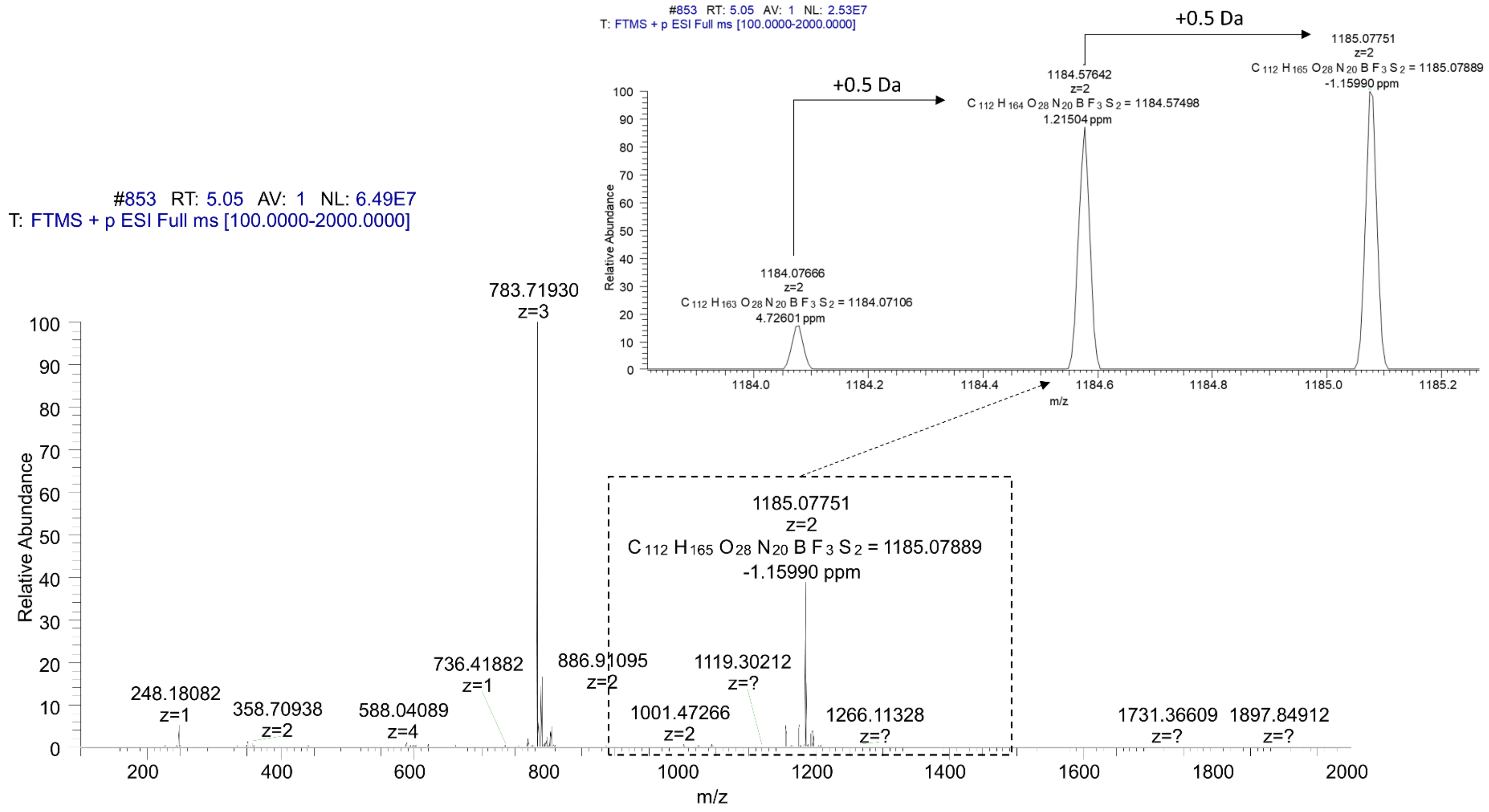

Figure S19. UHPLC-HRMS extracted ion chromatogram of $AmBF_3$-PEG_{11}-mTOC (**7**) DHP with a charge of Z=2 and a retention time of t_R=5.05 min in crude reaction mixture after IEDDA cycloaddition between **2** with **4**. Compound **7** was detected as doubly charged $[M+2H]^{2+}$ ion with mass error of Δ=1.21504 ppm (calculated *m/z* 1184.57498 for; $C_{112}H_{164}O_{28}N_{20}BF_3S_2^{2+}$, meas; *m/z* 1184.57642*).* Isotopic peak pattern of 0.5 Da indicates the peptide is detected in charge state +2.

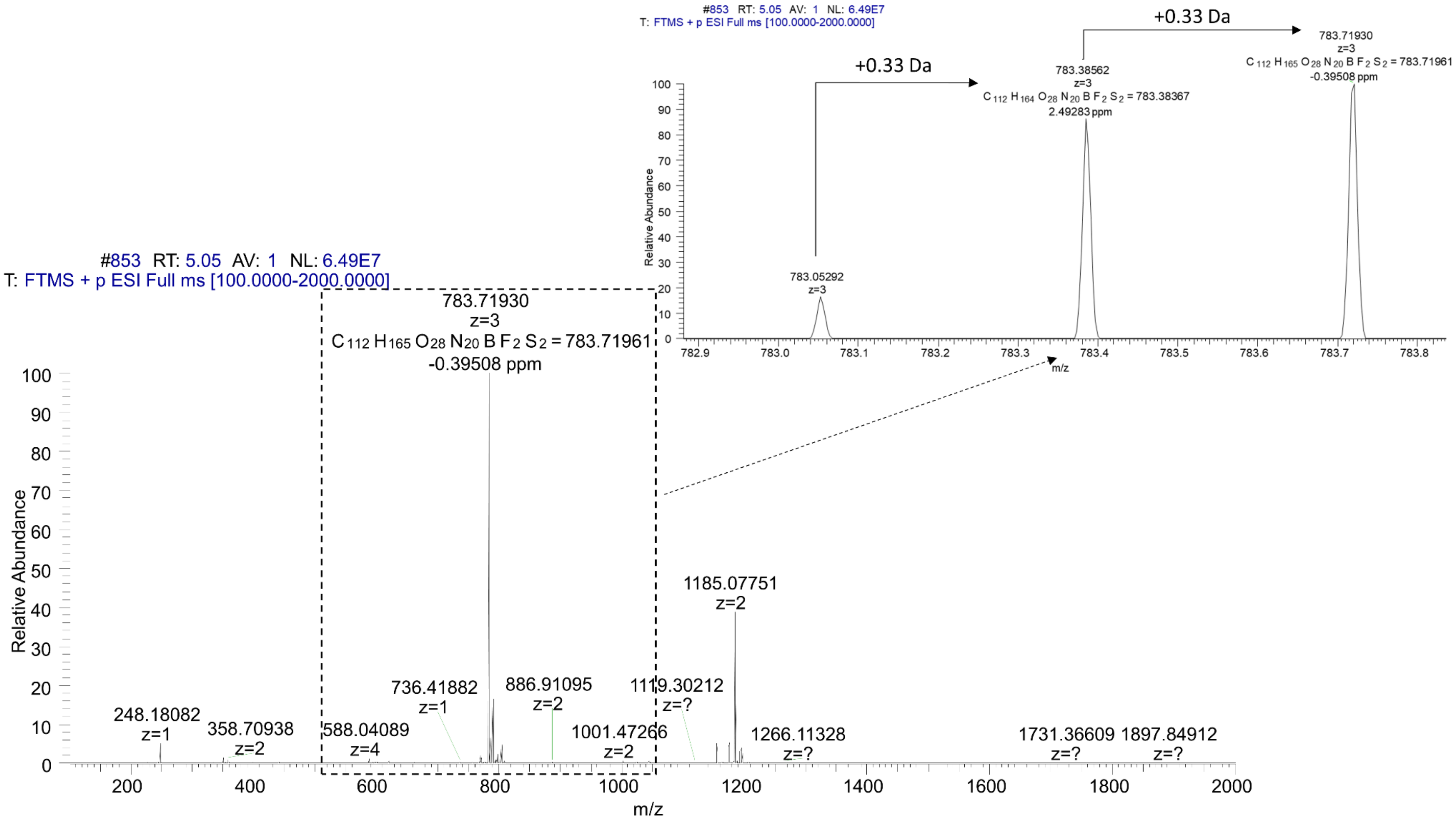

Figure S20. UHPLC-HRMS extracted ion chromatogram of AmBF$_3$-PEG$_{11}$-mTOC (**7**) DHP with a charge of Z=3 and a retention time of t_R=5.05 min in crude reaction mixture after IEDDA cycloaddition between **2** with **4**. Compound **7** was detected as triply charged $[M+3H-F]^{3+}$ ion with mass error of Δ=2.49283 ppm (calculated *m/z* 783.38367 for; $C_{112}H_{164}O_{28}N_{20}BF_2S_2^{3+}$, meas; *m/z* 783.38562*).* Isotopic peak pattern of 0.33 Da indicates the peptide is detected in charge state +3.

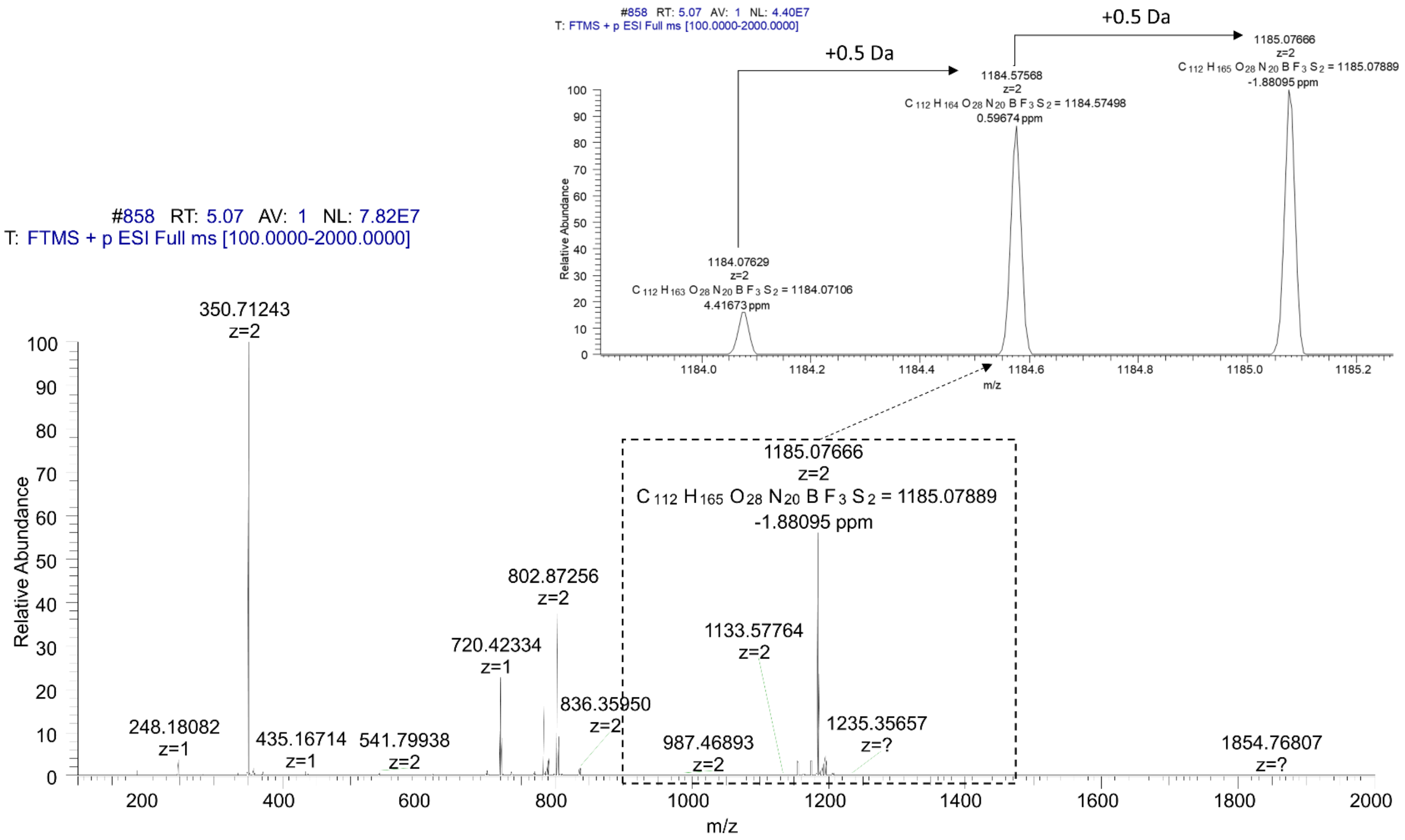

Figure S21. UHPLC-HRMS extracted ion chromatogram of AmBF$_3$-PEG$_{11}$-mTOC (**7**) DHP with a charge of Z=2 and a retention time of t$_R$=5.07 min in crude reaction mixture after IEDDA cycloaddition between **2** with **4**. Compound **7** was detected as doubly charged $[M+2H]^{2+}$ ion with mass error of Δ=0.59674 ppm (calculated *m/z* 1184.57498 for; $C_{112}H_{164}O_{28}N_{20}BF_3S_2^{2+}$, meas; *m/z* 1184.57568*).* Isotopic peak pattern of 0.5 Da indicates the peptide is detected in charge state +2.

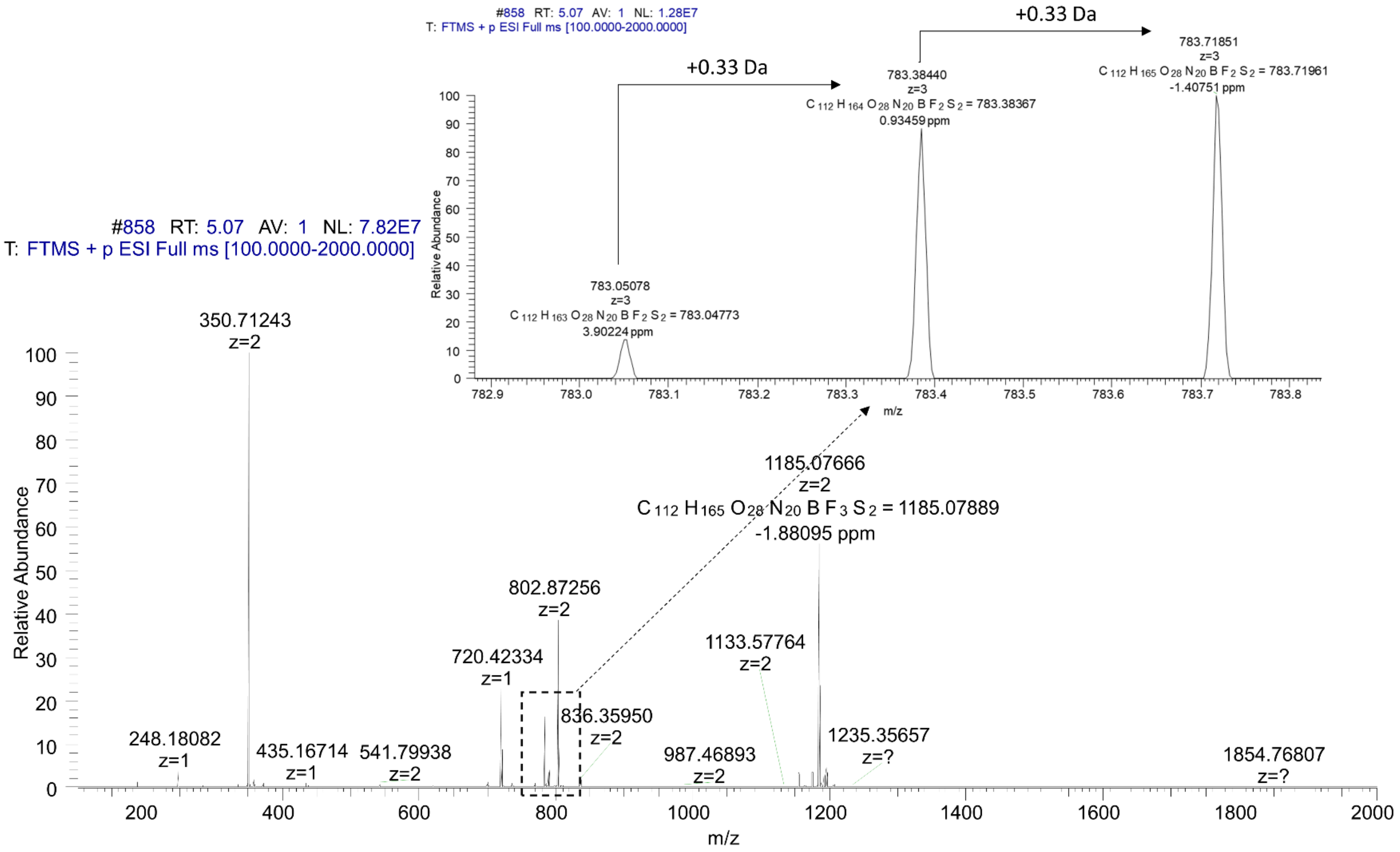

Figure S22. UHPLC-HRMS extracted ion chromatogram of AmBF$_3$-PEG$_{11}$-mTOC (**7**) DHP with a charge of Z=3 and a retention time of t_R=5.07 min in crude reaction mixture after IEDDA cycloaddition between **2** with **4**. Compound **7** was detected as triply charged $[M+3H-F]^{3+}$ ion with mass error of Δ=0.93459 ppm (calculated *m/z* 783.38367 for; $C_{112}H_{164}O_{28}N_{20}BF_2S_2^{3+}$, meas; *m/z* 783.38440*).* Isotopic peak pattern of 0.33 Da indicates the peptide is detected in charge state +3.

Table S7. Incubation tests of IEDDA cycloaddition product.

**Peptide**	**Peptide (nmol)**	**Solvent (v/v-%)**	**Vol. (µL)**	**pH**	**Temp. (°C)**	**Time (min)**	**[^{18}F] AmBF$_3$ (ox.)-TOC (%)**
[^{18}F]**6**	5	10 x PBS	180	6.8	**37**	10	5
[^{18}F]**6**	5	0.01 mM Citrate buffer	180	4	**37**	10	4
[^{18}F]**6**	5	0.15 mM Citrate buffer	180	3.75	**37**	10	5
[^{18}F]**6**	7.5	ACN:H_2O (25:75)	20	-	**40**	20	86
[^{18}F]**6**	2.5	0.15 mM Citrate buffer	180	3.75	**60**	10	9
[^{18}F]**6**	2.5	ACN:H_2O (25:75)	20	-	**60**	10	89
[^{18}F]**6**	2.5	ACN:H_2O (75:25)	20	-	**60**	10	59
[^{18}F]**6**	7.5	ACN:H_2O (13:87)	150	-	**60**	10	25
[^{18}F]**6**	2.5	ACN:H_2O (40:60)	25	-	**60**	15	77
[^{18}F]**6**	2	ACN:H_2O (25:75)	60	-	**60**	15	84
[^{18}F]**6**	2-50	ACN:H_2O (≥95% H_2O)	20-200	-	**60**	10	100
[^{18}F]**7**	12.5	ACN:H_2O (14:86)	56	-	**40**	10	65
[^{18}F]**7**	12.5	ACN:H_2O (14:86)	56	-	**50**	10	84
[^{18}F]**7**	12.5	ACN:H_2O (14:86)	56	-	**60**	10	100

References

1.Liu, Z.; Lin, K. S.; Benard, F.; Pourghiasian, M.; Kiesewetter, D. O.; Perrin, D. M.; Chen, X. One-step ^{18}F labeling of biomolecules using organotrifluoroborates. *Nat Protoc* **2015,** *10*, (9), 1423-32.

2. S. Otaru, A. Paulus, S. Imlimthan, I. Kuurne, H. Virtanen, H. Liljenbäck, T. Tolvanen, T. Auchynnikava, A. Roivainen, K. Helariutta, M. Sarparanta, A.J. Airaksinen, Development of [^{18}F]$AmBF_3$ Tetrazine for Radiolabeling of Peptides: Preclinical Evaluation and PET Imaging of [^{18}F]$AmBF_3$-PEG_7-Tyr^3-Octreotide in an AR42J Pancreatic Carcinoma Model, Bioconjugate Chemistry, **2022**,10.1021/acs.bioconjchem.2c00231.
